# Clustering Hybrid Functional and Vector Data

**DOI:** 10.1002/bimj.70184

**Published:** 2026-10

**Authors:** Sekha D. Daluwatumulle, Jeong Hoon Jang, Ying Cui, Amita K. Manatunga

**Affiliations:** 1 Department of Biostatistics and Bioinformatics, Emory University, Atlanta, Georgia, USA; 2 Department of Biostatistics and Data Science, University of Texas Medical Branch, Galveston, Texas, USA; 3 Department of Biomedical Data Science, Stanford University, Stanford, California, USA

**Keywords:** clustering, functional data, hybrid data, principal component analysis, unsupervised learning

## Abstract

Modern biomedical studies increasingly collect hybrid data, consisting of functional observations (e.g., curves or images) and multiple scalar (vector-valued) clinical variables. Integrating these modalities in an unsupervised learning framework can improve patient subgroup identification and interpretation, yet most existing clustering approaches handle only a single data type. We introduce a clustering framework for hybrid data combining multivariate functional and vector outcomes. The method first applies hybrid principal component analysis to capture joint mean structures and modes of variation across modalities, then iteratively reassigns subjects using a hybrid distance metric based on cluster-specific Karhunen–Loéve expansions. This approach accommodates multiple functions of varying dimensions, integrates contributions from functional and vector components in mean and covariance structures, and avoids parametric distributional assumptions. Simulation studies show that the proposed method outperforms competing approaches when subgroup information, in terms of mean and/or variance differences, is either distributed across modalities or concentrated in a single modality. We illustrate the method using renal radionuclide imaging data, including baseline and diuretic renogram curves and pharmacokinetic variables from patients with suspected kidney obstruction. The method identifies clinically meaningful subgroups consistent with expert assessment, demonstrating its potential to support data-driven decision-making in multimodal biomedical studies.

## 1 | Introduction

Recent advances in biomedical technology have enabled the collection of diverse data types, including scalars, vectors, tensors, and functions, from the same observational units. Integrating these data types can improve disease monitoring, diagnosis, and the study of underlying physiological mechanisms. However, combining multiple data modalities remains analytically challenging, and statistical methods for multimodal data integration are still relatively underdeveloped compared with methods for unimodal data.

Our work focuses on settings in which both functional and scalar outcomes are measured for each subject. In many biomedical studies, multiple functions and scalar variables are collected per subject, resulting in a hybrid data structure consisting of a functional component, comprising one or more functions, and a vector component, comprising scalar variables. Clustering such hybrid data can help uncover homogeneous subgroups that reflect underlying physiological processes or disease subtypes (Cohen et al., 2010; Paoletti et al., 2009). In this paper, we aim to cluster subjects into homogeneous groups based on joint functional and vector-valued outcomes, with the goal of supporting disease risk assessment and targeted clinical decision-making.

This work is motivated by a renal study conducted at Emory University. Radionuclide imaging plays a crucial role in the non-invasive evaluation of potential kidney obstruction and is integral to the clinical management of patients with suspected obstruction. Renal scans are performed following the intravenous injection of Tc-99m mercaptoacetyltriglycine (MAG3), which is rapidly cleared from the bloodstream by the kidneys and travels down the ureters to the bladder. Gamma photons emitted by the tracer are captured by a gamma camera and quantified for analysis (Taylor et al., 2008). For each kidney, two renogram curves are generated based on photon counts detected in the entire kidney: a baseline renogram curve, representing the time-varying MAG3 photon counts during the initial 24-min scanning period, and a diuretic renogram curve, representing the MAG3 photon counts during a second 20-min scanning period following an intravenous injection of furosemide, a potent diuretic. In addition, scalar pharmacokinetic variables summarizing renal excretory and drainage function are derived from the cortical renogram; the full list of variables is provided in Section 4.

Drawing on their clinical expertise in kidney imaging, radiologists interpret two complementary modalities of renogram data: renogram curves (baseline and diuretic; functional data) and scalar pharmacokinetic variables (vector data), in order to evaluate potential obstruction. Accurate interpretation of renogram data requires substantial expertise in renal physiology and MAG3 pharmacokinetics, and diagnostic performance varies widely, particularly in settings where radiologists with limited nuclear medicine training conduct most evaluations without expert input due to high scan volumes. Reported inter-reader discrepancies range from 9% to 72% (Jakšić et al., 2005; Taylor and Garcia, 2014). A major challenge is the absence of an established gold standard for obstruction diagnosis using radionuclide imaging, which contributes to interpretive variability. Given this context, a data-driven clustering algorithm that identifies homogeneous subgroups using both renogram curves and pharmacokinetic variables could support clinical interpretation and help identify clinically meaningful risk groups.

Importantly, the renal imaging application requires a clustering framework that can capture more than differences in average profiles. Clinically meaningful renal subgroups may differ not only in their mean renogram curve patterns and pharmacokinetic profiles, but also in their subgroup-specific modes of variability across these features. For example, obstructed and unobstructed kidneys may differ in mean uptake and clearance behavior, while also exhibiting different variation in timing, magnitude, clearance patterns, and scalar pharmacokinetic characteristics. Thus, a method that only compares mean curves, vectorizes functional observations, or applies standard clustering to global dimension-reduction scores may fail to capture clinically meaningful subgroup-specific mean-covariance structure. This motivates the need for a novel clustering framework that can jointly model functional and vector components through cluster-specific hybrid mean and covariance structures.

Although clustering methods have been extensively studied, most existing approaches focus on vector data consisting of multiple scalar variables. Comprehensive reviews of clustering methods for such vector data, including Gaussian model-based and nonparametric approaches, are provided by Nagpal et al. (2013) and Banfield and Raftery (1993). A widely used nonparametric method is K-means (Lloyd, 1982; MacQueen, 1967), which is popular due to its simplicity and scalability but can perform poorly in high-dimensional settings and is sensitive to outliers (Jie et al., 2020; Mittal et al., 2019). When applied to functional data, multivariate clustering methods often require vectorizing curves into longitudinal measurements, which can increase dimensionality and ignore the continuous structure of the data. This is particularly relevant for our renogram data, where features such as prolonged MAG3 accumulation and delayed elimination are inherently functional and may not be well represented by simple vectorization.

Unsupervised clustering methods tailored to functional data have also been extensively studied; see Jacques and Preda (2014) and Zhang and Parnell (2023) for comprehensive reviews and references therein. Among these, several model-based approaches have been developed (Luan and Li, 2003; Ray and Mallick, 2006; Leng and Müller, 2006; Schmutz et al., 2020; Gertheiss et al., 2024). For example, Luan and Li (2003) proposed a mixture model framework for clustering time-course gene expression data, Ray and Mallick (2006) introduced a wavelet-based model, Leng and Müller (2006) employed functional principal component-based logistic regression, and Schmutz et al. (2020) used latent mixture models to characterize group-specific functional sub-spaces. In a nonparametric framework, Chiou and Li (2007) proposed a method that clusters functional data by capturing both differences in mean structure and modes of variation across groups.

While these methods are effective for either functional or vector data, they are typically not designed to integrate both data modalities. The literature on clustering methods that jointly handle functional and vector data is quite limited. Wu and Li (2022) proposed a clustering technique for hybrid data combining functional and vector components. Specifically, Wu and Li developed a probabilistic model for clustering spatially correlated functional data alongside multiple scalar variables. However, this approach relies on parametric model assumptions, which can be difficult to specify correctly for complex and heterogeneous biomedical data. For instance, in our radionuclide imaging study, the pharmacokinetic variables exhibit highly skewed marginal distributions, making it challenging to specify a reasonable joint parametric distribution.

This paper addresses this methodological gap by proposing a nonparametric reconstruction-based clustering framework for hybrid functional/vector data. The main conceptual contribution is to formulate clustering for hybrid random objects through cluster-specific hybrid reconstructability. Specifically, each cluster is defined not as a centroid, not as a region in a global principal component score space, and not as a purely functional subspace, but as a distinct hybrid subprocess characterized by its own hybrid mean element, covariance operator, and low-rank Karhunen–Loéve (KL) representation in a product Hilbert space containing both functional and vector components. Under this formulation, each observation is assigned to the cluster under which it is most compatible with the cluster-specific hybrid mean-covariance structure, as measured by leave-one-out reconstruction error under the hybrid norm. Thus, the clustering target is shifted from proximity to global dimension-reduction scores to compatibility with a cluster-specific hybrid mean-covariance structure.

The proposed method leverages the key strengths of two important existing frameworks while reformulating them to address the scientific needs of the renal imaging application. Building on the hybrid PCA formulation of Jang (2021), we use the product Hilbert space representation to jointly encode functional and vector components. However, rather than using hybrid PCA as a final global dimension-reduction step, the proposed method uses the hybrid eigenspace representation as a building block for estimating local, cluster-specific hybrid eigenspaces that characterize subgroup-specific structure. Building on the KL-based functional clustering principle of Chiou and Li (2007), we extend the reconstruction-based assignment idea beyond purely functional observations by redefining the objects, mean structure, covariance operator, reconstruction loss, and reassignment criterion in a product Hilbert space containing both functional and vector components. Consequently, functional curves, images, scalar variables, and their joint modes of variation can all contribute to the cluster-specific reconstruction loss and reassignment criterion. To our knowledge, no existing nonparametric clustering framework has unified the hybrid representation of Jang (2021) and the reconstruction-based KL clustering principle of Chiou and Li (2007) to cluster hybrid functional/vector observations through subgroup-specific mean-covariance structures.

The resulting algorithm, referred to as HClust, proceeds iteratively. It first obtains initial cluster assignments using global hybrid principal component scores. It then repeatedly estimates cluster-specific hybrid mean and covariance structures, reconstructs each observation under each candidate cluster-specific hybrid KL expansion, and updates cluster membership according to the corresponding hybrid reconstruction error until convergence. This formulation allows subgroup differences to arise from functional features, vector features, or their joint covariance structure, while avoiding parametric distributional assumptions.

The remainder of the paper is organized as follows. Section 2 introduces the proposed methodology and the HClust algorithm. Section 3 presents simulation results comparing the performance of our method to existing approaches. In Section 4, we apply the proposed clustering method to renal study data. Finally, Section 5 offers concluding remarks and discusses the broader implications of our findings.

## 2 | Methods

### 2.1 | Random Hybrid Object

Let ${\left\{Y^{\left(k\right)}\right\}}_{k=1}^{K}$ denote a collection of real-valued random functions, where each $Y^{\left(k\right)}$ is defined on a compact domain ${𝓣}_{k}\subset R^{d}$; that is, $Y^{\left(k\right)}:{𝓣}_{k}\to R$. We assume that $Y^{\left(k\right)}\in L^{2}({𝓣}_{k})$, the Hilbert space of square integrable functions with respect to the Lebesgue measure $dt_{k}$ on ${𝓣}_{k}$. If ${𝓣}_{k}\subset R(d=1)$, then $Y^{\left(k\right)}$ is a one-dimensional function (curve), and its realized value at $t_{k}\in {𝓣}_{k}$ is denoted by $Y^{\left(k\right)}(t_{k})$. If ${𝓣}_{k}\subset R^{2}(d=2),Y^{\left(k\right)}$ is a two-dimensional function (image), and its realized value at $t_{k}=(u_{k},v_{k})\in {𝓣}_{k}$ is written as $Y^{\left(k\right)}(t_{k})$. Let $Y=(Y^{\left(1\right)},\ldots ,Y^{\left(K\right)})\in 𝓕=L^{2}({𝓣}_{1})\times \cdots L^{2}({𝓣}_{K})$ denote the random functional object that combines all K random functions. The realized version of the functional object Y at $t=(t_{1},\ldots ,t_{K})\in {𝓣}_{1}\times \cdots {𝓣}_{K}=𝓣$, that is, the collection of the realized values of the K functions at their respective domains, can be written as $Y(t)=(Y^{\left(1\right)}(t_{1}),\ldots ,Y^{\left(K\right)}(t_{K}))$. If K=1, each observational unit consists of a single function and is referred to as univariate functional data. If K≥2, each observational unit includes two or more functions and is referred to as multivariate functional data. Let $X=(X^{\left(1\right)},\ldots ,X^{\left(p\right)})$ denote a p-dimensional random vector in $R^{p}$, where each component $X^{\left(r\right)}(r=1,\ldots ,p)$ represents a scalar-valued random variable.

Let Z=(Y,X) denote a random hybrid object that combines functional and vector components into an ordered pair belonging to $𝓗=𝓕\times R^{p}$. Let Z[t]=(Y(t),X) denote the corresponding hybrid object with its functional component evaluated at $t=\left(t_{1},\ldots ,t_{K}\right)$. In our application, for each kidney, we observe Z=(Y,X), where $Y=(Y^{\left(1\right)},Y^{\left(2\right)})$ consists of baseline and diuretic renogram curves, and $X\in R^{6}$ represents six pharmacokinetic variables.

We define the inner product between two hybrid objects, $Z_{1}=\left(Y_{1},X_{1}\right)$ and $Z_{2}=\left(Y_{2},X_{2}\right)$, as the sum of the inner product in the 𝓕 space (denoted ${\left\langle \cdot ,\cdot \right\rangle }_{ℱ}$) and the inner product in the Euclidean space $R^{p}$ (denoted $\langle \cdot ,\cdot \rangle_{R^{p}}$) as follows:

(1) $${\left\langle Z_{1},Z_{2}\right\rangle }_{ℋ}={\left\langle Y_{1},Y_{2}\right\rangle }_{ℱ}+{\left\langle X_{1},X_{2}\right\rangle }_{R^{p}}\;\;=\sum_{k=1}^{K}\int_{{𝓣}_{k}}Y_{1}^{\left(k\right)}\left(t_{k}\right)Y_{2}^{\left(k\right)}\left(t_{k}\right)dt_{k}+\sum_{r=1}^{p}X_{1}^{\left(r\right)}X_{2}^{\left(r\right)}.$$

Based on this inner product, the distance metric between two hybrid objects can be written based on the corresponding norm as: ${\left\|Z_{1}-Z_{2}\right\|}_{ℋ}={\left\langle Z_{1}-Z_{2},Z_{1}-Z_{2}\right\rangle }_{𝓗}^{1/2}$. The hybrid space 𝓗, equipped with the inner product ${\left\langle \cdot ,\cdot \right\rangle }_{ℋ}$ defined in Equation (1), forms a separable Hilbert space (Conway, 1990). In this space, assuming $E\|Z{\|}_{ℋ}^{2}<\infty$, we define the mean and covariance operator of a random element Z=(Y,X)∈𝓗 as $\mu =E(Z)=\left(\mu_{Y},\mu_{X}\right)$, where $\mu_{Y}=E(Y)=(E(Y^{\left(1\right)}),\ldots ,E(Y^{\left(K\right)}))$ and $\mu_{X}=E(X)=(E(X^{\left(1\right)}),\ldots ,E(X^{\left(p\right)}))$, and define the covariance operator as Γ=E{(Z−μ)⊗(Z−μ)}, where Γ:𝓗→𝓗 is a linear operator induced by the tensor product ⊗.

### 2.2 | Hybrid Karhunen–Loéve (KL) Expansion and Principal Component Analysis

In this section, we review the concept of hybrid principal component analysis (HPCA),(Jang, 2021) which is based on the eigen-decomposition of a covariance operator Γ that captures the joint variation of functional and vector components. HPCA yields interpretable principal components that mirror the structure of each hybrid observation and produces a unified set of principal scores that serve as a low-dimensional representation of the combined functional and vector data.

The covariance operator Γ has been shown to be positive, self-adjoint, and compact under mild regularity conditions (see Theorem 1 in Jang (2021)). Therefore, by the Hilbert-Schmidt theorem (e.g., Theorem 4.2.4 in Hsing and Eubank (2015)), there exists a complete orthonormal system of eigenfunctions ${\left\{\xi_{m}\right\}}_{m\in N}\subset 𝓗$ and a corresponding sequence of non-increasing, non-negative eigenvalues ${\left\{\lambda_{m}\right\}}_{m\in N}$ (i.e., $\lambda_{1}\geq \lambda_{2}\geq \cdots \geq 0$) such that

$$\Gamma \xi_{m}=\lambda_{m}\xi_{m},\;\text{for}\;m\in N,$$

with lim_*m*→∞_ *λ*_*m*_ = 0. The corresponding spectral decomposition (e.g., Theorem 7.2.6 in Hsing and Eubank (2015)) is

$$\Gamma =\sum_{m=1}^{\infty }\lambda_{m}\left(\xi_{m}⊗\xi_{m}\right),$$

which leads to the following Karhunen-Loéve (KL) expansion (see Theorem 3 of Jang (2021)) of the hybrid object that serves as the foundation of HPCA:

(2) $$Z[t]=\mu [t]+\sum_{m=1}^{\infty }\rho_{m}\xi_{m}[t],\;t\in 𝓣.$$

The eigenvalues *λ_m_* quantify the amount of variability in the hybrid object **Z** explained by the corresponding eigenfunctions $\xi_{m}[t]=(\psi (t),\theta_{m})=(\psi_{m}^{\left(1\right)}(t_{1}),\ldots ,\psi_{m}^{\left(k\right)}(t_{k}),\theta_{m}^{\left(1\right)},\ldots \theta_{m}^{\left(p\right)})\in 𝓗$, also referred to as hybrid principal components (PCs). The first hybrid PC $\xi_{1}=(\psi_{1},\theta_{1})$ captures the direction of maximum joint variability between the functional and vector components, with $\psi_{1}$ representing the functional part and $\theta_{1}$ representing the vector part. Subsequent PCs capture orthogonal directions of decreasing variability. The random variables $\rho_{m}={\left\langle Z-\mu ,\xi_{m}\right\rangle }_{ℋ}$ have mean zero and variance *λ_m_*, and are referred to as hybrid PC scores. Note that the mean function takes the form of $\mu [t]=(\mu_{Y}(t),\mu_{X})$, where $\mu_{Y}(t)=(\text{E}\{Y^{\left(1\right)}(t_{1})\},\ldots ,\text{E}\{Y^{\left(K\right)}(t_{K})\}$ and $\mu_{X}=(\text{E}(X^{\left(1\right)}),\ldots ,\text{E}(X^{\left(p\right)}))$.

In practice, a truncated version of the KL expansion (2) is used:

(3) $$\tilde{Z}[t]=\mu [t]+\sum_{m=1}^{M}\rho_{m}\xi_{m}[t],\;t\in 𝓣.$$

The first M PC scores, $\left\{\rho_{1},\ldots ,\rho_{M}\right\}$ provide an optimal M-dimensional approximation of the infinite-dimensional hybrid object **Z**, in the sense that the corresponding PCs satisfy,

$${\left\{\xi_{m}\right\}}_{m=1}^{M}=arg\;\underset{{\left\{h_{m}\right\}}_{m=1}^{M}}{min}E{\left\|Z-\sum_{m=1}^{M}{\left\langle Z,h_{m}\right\rangle }_{ℋ}h_{m}\right\|}_{ℋ},$$

for a given M (Jang, 2021). These PC scores are thus often used as a lower-dimensional representation of **Z** for downstream clustering or regression analyses.

Next, our goal is to estimate the mean function, PCs (eigenfunctions), PC scores, and eigenvalues from the truncated KL expansion (3) using the observed data. These estimated quantities can then be used to reconstruct and predict the hybrid object through the expansion. Let ${\left\{Z_{i}\left[t_{i}\right]=(Y_{i}(t),X_{i})\right\}}_{i=1}^{n}$ denote a random sample of n observations, where each $Z_{i}\left[t_{i}\right]$ is a hybrid observation from the *i*th unit. The functional component of each observation is given by $Y_{i}(t_{i})=(Y_{i}^{\left(1\right)}(t_{i1}),\ldots ,Y_{i}^{\left(K\right)}(t_{iK}))$, where the *k*th element $Y_{i}^{\left(k\right)}(t_{ik})$ is observed at $J_{ik}$ time points $t_{ik}=(t_{ik1},\ldots ,t_{ikJ_{ik}})$ on a compact domain ${𝓣}_{k}$. For the k-th functional element, if $t_{ik}=t_{k}$ for all i=1,…,n, the corresponding functional data are referred to as regular functional data; otherwise, they are termed irregular functional data. The vector component $X_{i}=(X_{i}^{\left(1\right)},\ldots ,X_{i}^{\left(p\right)})$ consists of p scalar-valued observations for the *i*th unit.

We first consider the estimation of the mean function $\mu =\left(\mu_{Y},\mu_{X}\right)$. Firstly, the vector component $\mu_{X}$ can be estimated by the method of moments (i.e., sample mean function): ${\hat{\mu }}_{X}=\bar{X}=n^{-1}\sum_{i=1}^{n}X_{i}=(n^{-1}\sum_{i=1}^{n}X_{i}^{\left(1\right)},\ldots ,n^{-1}\sum_{i=1}^{n}X_{i}^{\left(p\right)})$. Secondly, for estimating the functional component $\mu_{Y}$, different strategies are used depending on whether the *k*th element $Y^{\left(k\right)}$ is regular or irregular. For regular elements, the mean function $\mu_{Y}^{\left(k\right)}=E(Y^{\left(k\right)})$ can be estimated using the pointwise sample mean: ${\hat{\mu }}_{Y}^{\left(k\right)}={\bar{Y}}^{\left(k\right)}(t)=n^{-1}\sum_{i=1}^{n}Y_{i}^{\left(k\right)}(t)$. For irregular elements, two approaches are commonly used. The first approach is to smooth each individual curve and then compute the pointwise sample mean across subjects (Ramsay and Dalzell, 1991). The second approach is to apply local linear regression directly to the pooled data (Chiou et al., 2014; Fan and Gijbels, 1996). For example, suppose $Y^{\left(k\right)}$ is observed on a one-dimensional domain. The mean function at time t, $\mu_{Y}^{\left(k\right)}(t)$, can be estimated from the pooled data $\left\{(t_{ikj},Y_{i}^{\left(k\right)}\left(t_{ikj}\right))i=1,\ldots ,n;j=1,\ldots ,J_{ik}\right\}$ as ${\hat{\mu }}_{Y}^{\left(k\right)}(t)=\hat{a}$ by solving the following weighted least squares problem:

$$(\hat{a},\hat{b})=arg\;\underset{a,b}{min}\sum_{i=1}^{n}\sum_{j=1}^{J_{ik}}{\left\{Y_{i}^{\left(k\right)}\left(t_{ikj}\right)-a-b\left(t_{ikj}-t\right)\right\}}^{2}K_{h}\left(t_{ikj}-t\right),$$

where $K_{h}(\cdot )$ is a symmetric kernel function with support [−1, 1] and bandwidth h>0. If $Y^{\left(k\right)}$ is observed on a two-dimensional domain, a similar but extended approach can be used by applying a local linear plane smoother to the pooled data (Chiou et al., 2014). Once the mean functions of all individual functional elements are estimated, they can be combined to set ${\hat{\mu }}_{Y}=({\hat{\mu }}_{Y}^{\left(1\right)},\ldots ,{\hat{\mu }}_{Y}^{\left(K\right)})$.

The PCs, PC scores, and eigenvalues can be estimated using the HPCA framework introduced by Jang (2021). Specifically, the framework first performs multivariate functional principal component analysis (MFPCA) on the functional component and classical principal component analysis (PCA) on the vector component, and then combines their respective PC representations to produce the hybrid counterparts. The key steps are briefly summarized as follows:

1. Perform MFPCA (Chiou et al., 2014; Happ and Greven, 2018) on the observed functional data $Y_{i}=(Y_{i}^{\left(1\right)},\ldots ,Y_{i}^{\left(K\right)}),i=1,\ldots ,n$, to estimate the functional PCs and PC scores-that is, obtain ${\hat{\phi }}_{l}=({\hat{\phi }}_{l}^{\left(1\right)},\ldots ,{\hat{\phi }}_{l}^{\left(K\right)})\in 𝓕$ and ${\hat{\zeta }}_{il}={\left\langle Y_{i}-{\hat{\mu }}_{Y},{\hat{\phi }}_{l}\right\rangle }_{F},l=1,\ldots ,L$.
2. Perform classical PCA(Anderson, 1984) on the observed vector data $X_{i},i=1,\ldots ,n$, to estimate vector PCs and vector PC scores—that is, obtain ${\hat{w}}_{h}\in R^{p}$ and ${\hat{\gamma }}_{ih}={\left\langle X_{i}-{\hat{\mu }}_{X},{\hat{w}}_{h}\right\rangle }_{R^{p}}$, h=1,…,H.
3. For each i, define the concatenated score vector ${\hat{\chi }}_{i}={\left[{\hat{\zeta }}_{i1},\ldots ,{\hat{\zeta }}_{iL},{\hat{\gamma }}_{i1},\ldots ,{\hat{\gamma }}_{iH}\right]}^{T}\in R^{L+H}$ and compute the sample covariance matrix $\hat{V}=(n-1{)}^{-1}\sum_{i=1}^{n}{\hat{\chi }}_{i}{\hat{\chi }}_{i}^{T}$.
4. Perform eigen-decomposition of $\hat{V}$ to obtain eigenvalues ${\hat{\lambda }}_{m}$ and orthonormal eigenvectors ${\hat{e}}_{m}=[{\hat{c}}_{m},{\hat{d}}_{m}]\in R^{L+H}$ for m=1,…,M, with M≤L+H, where ${\hat{c}}_{m}={\left[{\hat{c}}_{m1},\ldots ,{\hat{c}}_{mL}\right]}^{T}$ and ${\hat{d}}_{m}={\left[{\hat{d}}_{m1},\ldots ,{\hat{d}}_{mH}\right]}^{T}$.
5. The functional and vector parts of the m-th hybrid PC $\xi_{m}=(\psi_{m},\theta_{m})$ and the corresponding hybrid PC score for the i-th observation are given by:
$${\hat{\psi }}_{m}(t)=\sum_{l=1}^{L}{\hat{c}}_{ml}{\hat{\phi }}_{l}(t),\;{\hat{\theta }}_{m}=\sum_{h=1}^{H}{\hat{d}}_{mh}{\hat{w}}_{h},\;\text{and}\;{\hat{\rho }}_{im}=\sum_{l=1}^{L}{\hat{\zeta }}_{il}{\hat{c}}_{ml}+\sum_{h=1}^{H}{\hat{\gamma }}_{ih}{\hat{d}}_{mh}.$$

The truncation lags L,H, and M representing the number of components retained from MFPCA, PCA, and HPCA respectively, can be selected based on the proportion of variance explained. For example, one may choose $M={min}_{m}\{(\sum_{u=1}^{m}{\hat{\lambda }}_{u}/\sum_{u=1}^{n-1}{\hat{\lambda }}_{u})>\delta_{0}\}$, so that the first M hybrid PCs explain at least $100\delta_{0}\%$ of the total variability.

The low-rank structure learned through HFPCA provides a compact yet expressive representation of hybrid data, enabling efficient reconstruction of observed objects and accurate prediction of new hybrid observations. Once the hybrid PCs and their corresponding scores are estimated, the object can be approximated using the truncated KL expansion (3) as follows:

$$\hat{Z}[t]=\hat{\mu }[t]+\sum_{m=1}^{M}{\hat{\rho }}_{m}{\hat{\xi }}_{m}[t],\;t\in 𝓣.$$

This representation can be used to reconstruct previously observed data or predict unseen hybrid objects from their low-dimensional projections.

### 2.3 | Clustering Framework via Cluster-Specific KL Expansion for Hybrid Data

The goal of clustering methods is to identify homogeneous subgroups within a population. To this end, assume that **Z**[**t**] comprises subprocesses, each corresponding to a cluster indexed by a random variable S. That is, the cluster membership of each individual observation $Z_{i}$ is a categorical random variable taking values c∈{1,…,C} with unknown probability mass function $p_{S}(c)$. The subprocess associated with each cluster c is characterized by its own mean function $\mu^{\left[c\right]}$ and covariance operator $\Gamma^{\left[c\right]}$. These give rise to a cluster-specific KL expansion and its truncated version, analogous to Equations (2) and (3), based on cluster-specific eigenvalue-eigenfunction pairs $\left(\lambda_{m}^{\left[c\right]},\xi_{m}^{\left[c\right]}\right)$ of $\Gamma^{\left[c\right]}$. Specifically, for the i-th hybrid object in cluster c, denoted $Z_{i}^{\left[c\right]}$, the cluster-specific full and truncated KL expansions are given by:

(4) $$Z_{i}^{\left[c\right]}[t]=\mu^{\left[c\right]}[t]+\sum_{m=1}^{\infty }\rho_{im}^{\left[c\right]}\xi_{m}^{\left[c\right]}[t],\;\text{and}\;{\tilde{Z}}_{i}^{\left[c\right]}[t]=\mu^{\left[c\right]}[t]+\sum_{m=1}^{M_{c}}\rho_{im}^{\left[c\right]}\xi_{m}^{\left[c\right]}[t],$$

where $\rho_{im}^{\left[c\right]}={\left\langle Z_{i}^{\left[c\right]}-\mu^{\left[c\right]},\xi_{m}^{\left[c\right]}\right\rangle }_{ℋ}$ is a cluster-specific score.

Given an observed hybrid object $Z_{i}$, if its true cluster membership is c, then the reconstructed object ${\tilde{Z}}_{i}^{\left[c\right]}$ based on the cluster-specific truncated KL expansion (4) should closely approximate $Z_{i}$. Conversely, if $Z_{i}$ originates from a different cluster, the discrepancy between $Z_{i}$ and ${\tilde{Z}}_{i}^{\left[c\right]}$ will be larger. This idea parallels and extends the approach of Chiou and Li (2007), who proposed a functional clustering method based on the distance between the observed function and its cluster-specific KL-based prediction. While their method uses a norm defined on the functional domain, we adopt a hybrid norm throughout this work. Specifically, given an observed hybrid object $Z_{i}$ and the reconstructed cluster-specific object ${\tilde{Z}}_{i}^{\left[c\right]}$, we assign cluster membership according to the criterion:

(5) $$g_{i}={argmin}_{c\in \{1,\ldots ,C\}}{\left\|Z_{i}-{\tilde{Z}}_{i}^{\left[c\right]}\right\|}_{ℋ},$$

This distance-based criterion is used to reassign cluster labels at each iteration of the hybrid clustering algorithm, which we detail in Section 2.4.

### 2.4 | Clustering Algorithm

In this section, we introduce the algorithm for clustering hybrid data, along with its procedural steps. The number of clusters, C, must be specified prior to running the algorithm; a data-driven method for selecting C is presented in Section 2.6. Let ${\left\{Z_{i}=\left(Y_{i},X_{i}\right)\right\}}_{i=1}^{n}$ denote a random sample of n observations, where each $Z_{i}$ is a hybrid observation from the *i*th unit. The estimated mean of the hybrid data is denoted by $\hat{\mu }=\left({\hat{\mu }}_{Y},{\hat{\mu }}_{X}\right)$. The estimated *m*th eigenvalue and corresponding hybrid PC pair are denoted by ${\hat{\lambda }}_{m}$ and ${\hat{\xi }}_{m}=({\hat{\psi }}_{m},{\hat{\theta }}_{m})$ respectively. The estimated PC score for the *i*th observation is given by ${\hat{\rho }}_{im}={\left\langle Z_{i}-\hat{\mu },{\hat{\xi }}_{m}\right\rangle }_{ℋ}$, where the inner product (integral) is computed using numerical approximation methods. These estimates are obtained using the procedures described in Section 2.2. Let $g_{i}^{\left(w\right)}\in \{1,\ldots ,C\}$ denote the cluster label assigned to $Z_{i}$ at the *w*th iteration. The steps of the algorithm are provided in Algorithm 1.

We now present a supplementary explanation of each step of Algorithm 1. After appropriate scaling, the algorithm begins by applying HPCA to the full dataset to extract the first M leading hybrid PC scores ${\hat{\rho }}_{i1},\ldots ,{\hat{\rho }}_{iM}$ for each subject, where M is chosen such that the first M PCs explain approximately 95% or more of the total variability. A multivariate clustering method—K-means in this work—is then applied to these scores to obtain the initial cluster assignments $g_{i}^{\left(0\right)}$ for all i∈{1,…,n}.

The algorithm then proceeds with iterative cluster reassignment. Without loss of generality, suppose the cluster label for the *i*th observation $Z_{i}$ from the (w−1)th iteration is given as $g_{i}^{\left(w-1\right)}$. As described in Algorithm 1, the goal is to update the cluster assignment for $Z_{i}$ at the *w*th iteration. The procedure begins by excluding the *i*th hybrid observation from its current cluster. This exclusion avoids bias in predicting $Z_{i}$ using a model trained on data that includes itself, which could otherwise lead to overfitting and optimistic assignment. After this exclusion, we compute the cluster-specific estimates of the mean and PCs

$${\hat{\mu }}_{\left(-i\right)}^{\left[c\right]}=({\hat{\mu }}_{Y(-i)}^{\left[c\right]},{\hat{\mu }}_{X(-i)}^{\left[c\right]})\;\text{and}\;{\hat{\xi }}_{m(-i)}^{\left[c\right]}=({\hat{\psi }}_{m(-i)}^{\left[c\right]},{\hat{\theta }}_{m(-i)}^{\left[c\right]}),$$

by applying HPCA to all observations assigned to cluster c at iteration w−1, excluding *i*th observation (i.e., those with $g_{i^{\prime }}^{\left(w-1\right)}=c,i^{\prime }\neq i$). The subscript (−i) and superscript [c] indicate that the estimates are based on the sample from cluster *c* after removing the *i*th observation.

**ALGORITHM 1 |:** Hybrid clustering algorithm (HClust).

1. Initial step: Initial cluster assignment (i) Given the number of clusters C, apply K-means clustering to the first M leading hybrid PC scores ${\hat{\rho }}_{i1},\ldots ,{\hat{\rho }}_{iM}$, for i=1,…,n, obtained by applying HPCA to all observations (Jang, 2021).
2. Iteration steps: Update cluster membership $g_{i}^{\left(w\right)}$ at each iteration *w* (i) Leave out the *i*th observation **Z***_i_* (ii) For each cluster c∈{1,…,C}, perform HPCA on the observations belonging to cluster c, excluding the *i*th observation (i.e., those with $g_{i^{\prime }}^{\left(w-1\right)}=c$, for $i^{\prime }\neq i$), to obtain $${\hat{\mu }}_{\left(-i\right)}^{\left[c\right]}=({\hat{\mu }}_{Y\left(-i\right)}^{\left[c\right]},{\hat{\mu }}_{X\left(-i\right)}^{\left[c\right]}),$$ $${\hat{\xi }}_{m(-i)}^{\left[c\right]}=({\hat{\psi }}_{m(-i)}^{\left[c\right]},{\hat{\theta }}_{m(-i)}^{\left[c\right]}),$$ which are the leave-one-out estimates of the cluster-specific mean and PCs. (iii) For each cluster *c*, predict the hybrid object for observation *i* as: $${\hat{Z}}_{i}^{\left[c\right]}[t]={\hat{\mu }}_{\left(-i\right)}^{\left[c\right]}[t]+\sum_{m=1}^{M^{\left[c\right]}}{\hat{\rho }}_{im}^{\left[c\right]}{\hat{\xi }}_{m(-i)}^{\left[c\right]}[t],$$ where ${\hat{\rho }}_{im}^{\left[c\right]}={\left\langle Z_{i}-{\hat{\mu }}_{\left(-i\right)}^{\left[c\right]},{\hat{\xi }}_{m(-i)}^{\left[c\right]}\right\rangle }_{ℋ}$ (iv) Update the cluster membership of $Z_{i}$ as: $$g_{i}^{\left(w\right)}=arg\;{min}_{c\in \{1,\ldots ,C\}}{\left\|Z_{i}-{\hat{Z}}_{i}^{\left[c\right]}\right\|}_{ℋ}.$$ (v) Repeat steps (i)–(iv) until no further reclassifications occur.

Next, a prediction for the *i*th observation is constructed for each cluster c∈{1,…,C} by plugging the previously obtained leave-*i*-out cluster-specific mean and PC estimates, ${\hat{\mu }}_{\left(-i\right)}^{\left[c\right]}$ and ${\hat{\xi }}_{m(-i)}^{\left[c\right]}$, into the cluster-specific truncated KL expansion (4):

(6) $${\hat{Z}}_{i}^{\left[c\right]}[t]={\hat{\mu }}_{\left(-i\right)}^{\left[c\right]}[t]+\sum_{m=1}^{M^{\left[c\right]}}{\hat{\rho }}_{im}^{\left[c\right]}{\hat{\xi }}_{m(-i)}^{\left[c\right]}[t],$$

where $M^{\left[c\right]}$ is the number of hybrid PCs that adequately explain the total variability in cluster c, which may vary by iteration w and cluster. Here, the cluster-specific PC scores for the *i*th observation ${\hat{\rho }}_{im}^{\left[c\right]}$ are computed as ${\hat{\rho }}_{im}^{\left[c\right]}={\left\langle Z_{i}-{\hat{\mu }}_{\left(-i\right)}^{\left[c\right]},{\hat{\xi }}_{m(-i)}^{\left[c\right]}\right\rangle }_{ℋ}$.

The updated cluster membership $g_{i}^{\left(w\right)}$ is then assigned as the cluster that minimizes the distance between the observed hybrid object $Z_{i}$ and its cluster-specific predicted version ${\hat{Z}}_{i}^{\left[c\right]}$ across all clusters, according to the criterion (5) established in Section 2.3:

(7) $$g_{i}^{\left(w\right)}={\mathrm{argmin}}_{c\in \{1,\ldots ,C\}}\|Z_{i}-{\hat{Z}}_{i}^{\left[c\right]}\|.$$

The same procedure is applied to all i∈{1,…,n}, and cluster labels are updated iteratively. The algorithm terminates at the iteration in which no further reclassification occurs. We refer to this procedure as HClust throughout the paper.

A potential challenge in implementing Algorithm 1 is that the functional and vector components may differ in their units and variability. A sensible approach is to scale the vector part so that $Z=\left(Y,\omega^{1/2}X\right)$, where ω is used to balance the contributions of the functional and vector parts in the algorithm. Among the available choices, there are three commonly used approaches for selecting ω. The first approach, suggested by Ramsay and Silverman (2005) for univariate functional data and vector data, is to set ω=|𝓣|, where |𝓣| denotes the length of the functional domain. This choice gives differences in the functional component approximately the same weight as differences of similar magnitude in a single component of the vector part. Thus, it is most appropriate when the functional and vector components measure comparable quantities. If the two components measure fundamentally different quantities, however, balancing them through this fixed domain-length scaling may not be meaningful. The second approach, also suggested by Ramsay and Silverman (2005) for univariate functional data and vector data, is to use ω=|𝓣|/p, which gives differences in the functional component approximately the same weight as differences across all p components of the vector part. Although this approach accounts for the dimension of the vector component, it does not account for potentially different levels of observed variability between the functional and vector components.

The third approach is analogous to performing PCA based on a correlation matrix rather than a covariance matrix. Under this approach, the weight is chosen so that the total variability contributed by the functional component equals the weighted total variability contributed by the vector component. Specifically, differences in scale within each component are first addressed: the functional elements $Y_{i}=(Y_{i}^{\left(1\right)},\ldots ,Y_{i}^{\left(K\right)})$ can be rescaled to have unit integrated variance, and the vector elements $X_{i}=(X_{i}^{\left(1\right)},\ldots ,X_{i}^{\left(p\right)})$ can be standardized to have unit variance. Second, to balance variability between the functional and vector components, we derive a data-driven weight that reflects their relative total variability:

(8) $$\omega =\frac{\sum_{i=1}^{n}{\left\|Y_{i}(t)-{\hat{\mu }}_{Y}(t)\right\|}_{F}^{2}}{\sum_{i=1}^{n}{\left\|X_{i}-{\hat{\mu }}_{X}\right\|}_{R^{p}}^{2}}.$$

In our real-data analysis, we adopted this third weighting approach because it is more generally applicable when the functional and vector components are measured on different scales or exhibit different levels of variability. In our renal imaging application, the functional and vector components are both derived from the same underlying MAG3 renography process, but they represent different types of information and exhibit different levels of variation. Thus, balancing their contributions according to their observed total variability is more appropriate than using a fixed weight based only on the length of the functional domain or the number of vector components.

### 2.5 | Theoretical and Practical Interpretation of the Reassignment Criterion

We provide brief theoretical insight into HClust by leveraging the theoretical discussion and results in Section 3 and Theorem 1 of Chiou and Li (2007). We first discuss why clustering marginal HPCA scores, as obtained from the hybrid PCA framework of Jang (2021), may work reasonably well when clusters mainly differ in mean structure, but may be insufficient when clusters differ in their cluster-specific eigenspaces or modes of variation. This also explains why cluster-specific hybrid prediction and reassignment can improve subgroup identification. Let $Z_{c}=\left(Y_{c},X_{c}\right)$ denote a hybrid random object drawn from cluster *c*. Let μ and ${\left\{\xi_{j}\right\}}_{j\geq 1}$ denote the marginal hybrid mean and eigenfunctions from the pooled hybrid process, and let $\mu^{\left[c\right]}$, ${\left\{\xi_{m}^{\left[c\right]}\right\}}_{m\geq 1}$, and ${\left\{\lambda_{m}^{\left[c\right]}\right\}}_{m\geq 1}$ denote the corresponding cluster-specific hybrid quantities. The marginal hybrid PC score for $Z_{c}$, given by $\rho_{j}\left(Z_{c}\right)={\left\langle Z_{c}-\mu ,\xi_{j}\right\rangle }_{ℋ}$, satisfies $E\left\{\rho_{j}\left(Z_{c}\right)\right\}={\left\langle \mu^{\left[c\right]}-\mu ,\xi_{j}\right\rangle }_{ℋ}$ and $cov\left\{\rho_{j}\left(Z_{c}\right),\rho_{k}\left(Z_{c}\right)\right\}=\sum_{m=1}^{\infty }\lambda_{m}^{\left[c\right]}{\left\langle \xi_{m}^{\left[c\right]},\xi_{j}\right\rangle }_{ℋ}{\left\langle \xi_{m}^{\left[c\right]},\xi_{k}\right\rangle }_{ℋ}$.

The first expression shows that mean differences between $\mu^{\left[c\right]}$ and μ are reflected in the expected marginal HPCA scores, $E\left\{\rho_{j}\left(Z_{c}\right)\right\}$, which explains why marginal HPCA score clustering can be effective when subgroup separation is driven primarily by mean structure. However, clustering marginal HPCA scores alone may not adequately distinguish subgroups with different covariance structures or joint functional-vector modes of variation. Such differences are reflected in the within-cluster covariance of the marginal HPCA scores, $cov\left\{\rho_{j}\left(Z_{c}\right),\rho_{k}\left(Z_{c}\right)\right\}$, only through the alignment between the cluster-specific eigenfunctions $\xi_{m}^{\left[c\right]}$ and the marginal eigenfunctions $\xi_{j}$. If the cluster-specific eigenspaces are poorly aligned with the retained marginal HPCA directions, the subgroup-specific covariance structure may be obscured in the marginal score space. This motivates the prediction-based reassignment step in HClust through the prediction criterion in Equation (6) and the clustering criterion in Equation (7), in which candidate cluster structures are compared according to how well each cluster-specific hybrid mean-covariance structure reconstructs the observed hybrid object, thereby incorporating both mean structure and modes of variation.

We next discuss identifiability conditions under which cluster membership can be distinguished via the prediction criterion in Equation (6) and the clustering criterion in Equation (7), based on the estimated cluster structures. Let ${𝓢}_{M_{c}}^{\left[c\right]}=span\{\xi_{1}^{\left[c\right]},\ldots ,\xi_{M_{c}}^{\left[c\right]}\}$ denote the retained cluster-specific hybrid eigenspace for cluster *c*. For a hybrid object $Z_{c}$ drawn from cluster c, the reconstruction under its own cluster structure is ${\tilde{Z}}_{c}^{\left[c\right]}=\mu^{\left[c\right]}+\sum_{m=1}^{M_{c}}{\left\langle Z_{c}-\mu^{\left[c\right]},\xi_{m}^{\left[c\right]}\right\rangle }_{ℋ}\xi_{m}^{\left[c\right]}$, whereas the reconstruction under a competing cluster d is ${\tilde{Z}}_{c}^{\left[d\right]}=\mu^{\left[d\right]}+\sum_{\ell =1}^{M_{d}}{\left\langle Z_{c}-\mu^{\left[d\right]},\xi_{\ell }^{\left[d\right]}\right\rangle }_{ℋ}\xi_{\ell }^{\left[d\right]}$. Parallel to the identifiability discussion in Chiou and Li (2007), two cluster structures may be difficult to distinguish if the retained hybrid eigenspace of one cluster is contained in that of another, that is, ${𝓢}_{M_{c}}^{\left[c\right]}\subseteq {𝓢}_{M_{d}}^{\left[d\right]}$, and the corresponding hybrid mean structures are not separable, for example when $\mu^{\left[c\right]}=\mu^{\left[d\right]}$ or when $\mu^{\left[c\right]},\mu^{\left[d\right]}\in {𝓢}_{M_{d}}^{\left[d\right]}$. Under such non-identifiability conditions, the discrepancy ${\left\|{\tilde{Z}}_{c}^{\left[c\right]}-{\tilde{Z}}_{c}^{\left[d\right]}\right\|}_{ℋ}^{2}$ may be small, apart from truncation and estimation errors. Conversely, when these non-identifiability conditions do not hold, the hybrid reconstruction distance can reflect meaningful differences in cluster-specific hybrid mean elements, eigenspaces, or both. Therefore, the prediction criterion in Equation (6) and the clustering criterion in Equation (7) are expected to be informative when cluster-specific hybrid mean and covariance structures are distinguishable, the retained hybrid components capture dominant within-cluster variation, the functional and vector components are appropriately scaled, and clinically meaningful subgroups differ in functional features, vector features, or their joint modes of variation.

The above discussion provides practical guidance on when the reconstruction-based reassignment criterion is expected to be useful. When subgroup separation is primarily driven by mean differences that are well captured by the leading global HPCA scores, the initial HPCA/K-means step may already perform well, and the reconstruction-based reassignment step may provide limited additional improvement. In contrast, when clusters differ through subgroup-specific eigenspaces or modes of variation, global HPCA score clustering may obscure cluster-specific covariance structure because it represents all observations using a single pooled eigenspace. The reconstruction-based criterion instead compares each observation with candidate cluster-specific hybrid mean-covariance structures, and can therefore provide information beyond simple centroid separation when one cluster-specific structure reconstructs an observation substantially better than another.

The reconstruction-based criterion may be less informative in a special boundary case where two clusters share the same retained eigenspace and the mean separation lies entirely within that common retained eigenspace. For example, in a simple two-dimensional vector setting, suppose observations from cluster c follow $Z^{\left[c\right]}=\mu^{\left[c\right]}+\rho u+\epsilon ,c=1,2$, where u is a common dominant direction and only the first principal component is retained. If $\mu^{\left[1\right]}-\mu^{\left[2\right]}\in span(u)$, then the one-dimensional reconstruction projects observations onto the same direction along which the cluster means are separated. Consequently, the residual reconstruction error mainly reflects variation orthogonal to u, rather than the mean separation itself. Conversely, if the mean separation has a component outside the retained eigenspace, or if the clusters have distinct retained eigenspaces representing different modes of variation, reconstruction under an incorrect cluster structure tends to leave a larger residual. Thus, HClust should be viewed as combining an initial global HPCA/K-means step, which is useful for capturing simple mean-separated structure, with a cluster-specific reconstruction step, which is useful when subgroup differences involve distinguishable mean-covariance structures.

In practice, users can assess which situation is more relevant by examining several empirical diagnostics, including separation in the global HPCA score space, stability of the initial HPCA/K-means clustering, estimated cluster-specific mean functions and vector means, estimated cluster-specific eigenspaces, and reconstruction-error differences between candidate clusters. If the global HPCA scores show clear centroid separation and the cluster-specific reconstruction errors provide little additional separation, the initial HPCA/K-means clustering may be sufficient. In contrast, if cluster-specific mean functions, vector means, covariance structures, or eigenspaces differ meaningfully, and reconstruction-error differences clearly favor different cluster-specific models, then the full HClust reassignment step is likely to be informative. Stability across initializations or resampling, together with scientific interpretability of the resulting clusters, should also be considered.

### 2.6 | Data-Driven Framework for Selecting Number of Clusters

Determining the number of clusters is one of the major challenges in cluster analysis. Specifying too many clusters can lead to over-partitioning the data, resulting in convergence problems or interpretability issues. Conversely, specifying too few clusters may overlook important subgroups that could be associated with key mechanisms of disease or health conditions. In this work, we extend the “distortion function” approach (James and Sugar, 2003; Sugar and James, 2003), which has been successfully implemented in practice to guide the selection of the number of clusters, to a hybrid data setting. Given a cluster number C, we first define the distortion function as follows:

(9) $$d_{C}=\frac{1}{M}\underset{c_{1},\ldots ,C_{C}}{min}E\left\{{\left(\eta_{i}-C_{g_{i}}\right)}^{T}\Gamma^{-1}\left(\eta_{i}-C_{g_{i}}\right)\right\},$$

where $\eta_{i}$ denotes the lower-dimensional version of the hybrid data object $Z_{i},C_{g_{i}}$ is the closest cluster center to $\eta_{i}$ among $\left\{C_{1},\ldots ,C_{C}\right\}$, and M is the dimension of $\eta_{i}$. The covariance matrix Γ defines the metric for distance computation. We assume $\eta_{i}$ arises from an underlying mixture of C clusters, with the clusters identically distributed with covariance Γ. In our implementation, we set Γ=I. The distortion function basically measures how well data points fit within their respective clusters given cluster number C. A lower distortion function value typically indicates tighter, more cohesive clusters. The steps for determining the number of clusters using the distortion function are as follows:

1. Conduct HPCA on the full dataset ${\left\{Z_{i}\right\}}_{i=1}^{n}$ and obtain the PC scores for all observations. We then use these scores, $\eta_{i}=\left({\hat{\rho }}_{i1},\ldots ,{\hat{\rho }}_{iM}\right)$, as a lower-dimensional proxy to calculate the distortion function (9). Here, the number of PC scores for each subject, M, is chosen to explain a substantial proportion of the total variability; 99% is recommended and implemented in our paper.
2. Run the K-means algorithm on $\left\{\eta_{1},\ldots ,\eta_{n}\right\}$ for different numbers of clusters, C, and estimate the corresponding distortions as ${\hat{d}}_{C}=(Mn{)}^{-1}\sum_{i=1}^{n}{\left(\eta_{i}-C_{g_{i}}\right)}^{T}\left(\eta_{i}-C_{g_{i}}\right)$, where $C_{g_{i}}$ are the cluster centers identified by K-means.
3. Select a transformation power B>0 and calculate the ”jumps” in the transformed distortion as follows:
$$J_{C}=\left\{\begin{array}{ll} {\left\{{\hat{d}}_{C}\right\}}^{-B}-{\left\{{\hat{d}}_{C-1}\right\}}^{-B} & \text{for}\;C\geq 2;\\ {\left\{{\hat{d}}_{C}\right\}}^{-B} & \text{for}\;C=1. \end{array}\right.$$
  Each jump value $\left(J_{C}\right)$ quantifies the change in distortion when increasing the number of clusters from C−1 to C. Large jumps indicate substantial improvement in cluster separation when adding an additional cluster, while small jumps suggest diminishing returns from further partitioning.
4. Estimate the number of clusters as $C^{*}=arg\;{max}_{C}\left\{J_{C}\right\}$; that is, select the value of C corresponding to the largest jump, which reflects the most significant improvement in cluster separation.

The values of the “jumps” and, consequently, the selected number of clusters, can depend substantially on the choice of the transformation power B. The conventional approach in James and Sugar (2003) recommends choosing B as one half of the “effective” number of dimensions in the data. As an alternative to this potentially subjective choice, Cui et al. (2026) proposed a data-driven approach for determining the effective dimension used to select B. They also showed that the resulting procedure is robust to reasonable variation in the number of retained components M. We adapt this approach to the hybrid data setting by defining

$$b^{*}=min\left\{b\in \{1,\ldots ,M\}:\frac{\sum_{m=1}^{b}\tau_{m}}{\sum_{m=1}^{M}\tau_{m}}\geq \tilde{p}\right\},\;B=\frac{b^{*}}{2},$$

where $\tau_{1}\geq \cdots \geq \tau_{M}$ denote the eigenvalues of the covariance matrix of $\eta_{i}$, and $\tilde{p}=0.7$ or 0.8 is chosen to represent a moderate proportion of the variability in $\eta_{i}$.

Although this procedure provides a practical data-driven approach for selecting the number of clusters, several limitations should be noted. First, the distortion function is computed using the lower-dimensional HPCA score representation $\eta_{i}$, so its performance depends on whether the retained scores adequately capture the clustering structure in the original hybrid data. Second, the selected number of clusters may depend on the number of retained components *M*, the transformation power *B*, and the range of candidate values of *C* considered. Third, because the distortion is calculated using a K-means-type criterion with Γ = *I* in our implementation, the procedure is most directly aligned with compact, well-separated clusters in the HPCA score space. It may be less reliable when clusters are highly imbalanced, weakly separated, non-spherical, or primarily distinguished by cluster-specific covariance/eigenspace structures that are not well represented in the global HPCA score space. Therefore, the selected value of *C* should be considered together with clustering stability, interpretability, and scientific or clinical relevance.

## 3 | Simulation Studies

To evaluate the performance of our clustering algorithm, HClust, we conducted a series of simulation studies. We considered the following data-generating model to simulate hybrid objects, each consisting of both functional and vector components within the *c*th cluster:

(10) $$Z_{i}^{\left[c\right]}=(Y^{\left[c\right]},X^{\left[c\right]})=(F_{i}^{\left[c\right]}+W_{i}+\epsilon_{i},V_{i}^{\left[c\right]}+W_{i}),$$

where $F_{i}^{\left[c\right]}$ and $V_{i}^{\left[c\right]}$ denote the underlying cluster-specific functional and vector signals, respectively. The functional signal $F_{i}^{\left[c\right]}=(F_{i}^{\left(1)[c\right]},\ldots ,F_{i}^{\left(K)[c\right]})$ consists of K functions, where each $F_{i}^{\left(k)[c\right]}(k=1,\ldots ,K)$ is generated from a Gaussian process $GP(\mu_{F}^{\left(k)[c\right]},\Sigma^{\left(k)[c\right]})$ on a one-dimensional (d=1) discretized domain ${𝓣}_{\text{grid}}=\left\{\left(t^{\prime }-1\right)/59,t^{\prime }=1,\ldots ,60\right\}$, which consists of 60 equally spaced time points over the unit interval [0,1]⊂R. The cluster-specific mean function $\mu_{F}^{\left[c\right]}=(\mu_{F}^{\left(1)[c\right]},\ldots ,\mu_{F}^{\left(K)[c\right]})$ and covariance function $\Sigma {\left(s,t\right)}^{\left(k)[c\right]}$ are specified according to the simulation scenario. The independent functional measurement error term $\epsilon_{i}$ follows a multivariate normal distribution, $N_{60}\left(0,0.01\cdot I_{60}\right)$. The vector signal $V_{i}^{\left[c\right]}=(V_{i}^{\left(1)[c\right]},\ldots ,V_{i}^{\left(p)[c\right]})$ is generated from a multivariate normal distribution $N_{p}(\mu_{V}^{\left[c\right]},\alpha^{\left[c\right]}I_{p})$, where $\mu_{V}^{\left[c\right]}=(\mu_{V}^{\left(1)[c\right]},\ldots ,\mu_{V}^{\left(p)[c\right]})$ and $\alpha^{\left[c\right]}\in R$ denote the cluster-specific mean vector and variance parameter, respectively, both specified according to the simulation scenario. A subject-specific effect random effect $W_{i}∼N(0,0.1)$ is shared across both the functional and vector components, thereby inducing correlation both within and between components.

### 3.1 | Setup 1: Univariate Functional Data (K=1; d=1) and Four-Dimensional Vector Data (p=4)

We first consider the setting where each hybrid object consists of univariate functional data (K=1), and a 4-dimensional vector component (p=4). We generated data from two clusters c={1,2} under simulation scenarios defined by varying cluster-specific mean structures for the functional and vector signals, $\mu_{F}^{\left[c\right]}=\mu_{F}^{\left[1][c\right]}$ and $\mu_{V}^{\left[c\right]}=(\mu_{V}^{\left(1)[c\right]},\mu_{V}^{\left(2)[c\right]},\mu_{V}^{\left(3)[c\right]},\mu_{V}^{\left(4)[c\right]})$. We considered two levels of differences in the functional mean and covariance structures between clusters: large and small. For the large functional difference, we specified $\mu_{F}^{\left(1)[1\right]}(t)=0$ for all $t\in {𝓣}_{\text{grid}}$, $\mu_{F}^{\left(1)[2\right]}(t)=2{\left(t+0.5\right)}^{2}+t,\Sigma^{\left(1)[1\right]}(s,t)=e^{-2{\left(s-t\right)}^{2}}$, and $\Sigma^{\left(1)[2\right]}(s,t)=0.8^{\left|s-t\right|}/\left(1-0.8^{2}\right)$, for $s,t\in {𝓣}_{\text{grid}}$. For the small functional difference, we used $\mu_{F}^{\left(1)[1\right]}(t)=4{\left(t-0.5\right)}^{2},\mu_{F}^{\left(1)[2\right]}(t)=1.5e^{\left(-20{\left(t-0.2\right)}^{2}\right)}+1.5e^{\left(-40{\left(t-0.2\right)}^{2}\right)},\Sigma (s,t{)}^{\left(1)[1\right]}=e^{-2{\left(s-t\right)}^{2}}$, and $\Sigma {\left(s,t\right)}^{\left(1)[2\right]}=e^{-1.5{\left(s-t\right)}^{2}}$, for $s,t\in {𝓣}_{\text{grid}}$. We also considered two levels of differences in the vector mean and covariance structures between clusters: large and small. For the large difference, we used $\mu_{V}^{\left[1\right]}=0_{4\times 1},\mu_{V}^{\left[2\right]}=[20,20,50,50],\alpha^{\left[1\right]}=2$ and $\alpha^{\left[2\right]}=6$; for the small difference, we used $\mu_{V}^{\left[1\right]}=0_{4\times 1},\mu_{V}^{\left[2\right]}=[1,1,1,1],\alpha^{\left[1\right]}=2$ and $\alpha^{\left[2\right]}=5$.

Our proposed method, HClust, was applied to cluster the generated data using the weight specified in Equation (8), which balances the total variability contributed by the functional component with the weighted total variability contributed by the vector component. In addition, we considered several existing clustering methods for comparison. We first considered functional clustering techniques proposed by Chiou and Li (2007) and Schmutz et al. (2020). The method by Chiou and Li (2007) was applied using the FClust function available in the R package “fdaspace”, while the method by Schmutz et al. (2020) was implemented using the tfunHDDC function from the R package “tfunHDDC”. Since both FClust and tfunHDDC are designed exclusively for functional data, we applied them only to the functional component of the simulated hybrid data. We also considered the conventional K-means algorithm applied in two different ways. First, K-means was applied only to the vector component of the simulated data (denoted as K-means-Vector). Second, K-means was applied to both components simultaneously by first vectorizing the functional data and then concatenating it with the vector component to form a single feature vector (denoted as K-means-Both). Finally, we considered a method that first applies HPCA (Jang, 2021) and then performs clustering on the resulting scores. This approach corresponds to the initial clustering step used in Algorithm 1 of our proposed method.

Table 1 presents the clustering accuracy of the proposed HClust method and the competing methods across seven simulation scenarios, which represent small to large inter-cluster differences in the mean and covariance structures of the functional and vector components. The sample sizes for clusters 1 and 2 are balanced in each configuration, with 75, 100, or 125 subjects per cluster. The accuracy rates are defined as the proportions of correctly classified observations based on the known true cluster labels, averaged across 100 replicated datasets for each configuration. In scenario (i), where the inter-cluster differences are large in both the mean and covariance structures of the functional and vector components, all methods perform well, with HClust, FClust, K-means-Vector, and HPCA achieving accuracy rates close to 1. In scenario (ii), where the inter-cluster differences in the functional mean and covariance structures remain large but those in the vector component are small, HClust and FClust continue to achieve near-perfect accuracy, whereas K-means-Vector, which relies solely on the vector component, performs poorly. In scenario (iii), where the inter-cluster differences in the functional mean and covariance structures are small but those in the vector component are large, HClust, K-means-Vector, and HPCA achieve near-perfect accuracy, whereas the performance of methods that use only the functional component, namely FClust and tfunHDDC, declines substantially.

In scenario (iv), the inter-cluster mean differences are large in both the functional and vector components, whereas the inter-cluster covariance differences are small in both components. In this setting, HClust and HPCA achieve the best performance, suggesting that HClust, although it incorporates both mean and covariance differences, performs comparably to HPCA, which is specialized in detecting mean differences, as discussed in Section 2.4. In scenario (v), the inter-cluster mean differences are small in both the functional and vector components, while the functional component exhibits large inter-cluster differences in covariance structure and the vector component exhibits small inter-cluster covariance differences. In this setting, HClust outperforms the competing methods except for tfunHDDC. This may be because the greater between-cluster difference in functional variability generates more outlying functions in the simulated data, which may be particularly advantageous for tfunHDDC that does not rely on the KL-based prediction. In scenario (vi), where the inter-cluster differences arise primarily from the covariance structure of the vector component, HClust again outperforms the competing methods. Similar superior performance of HClust is observed in scenario (vii), where the inter-cluster differences are small in both the mean and covariance structures of both the functional and vector components, highlighting its ability to effectively integrate complementary information from both data types even when the individual signals are relatively weak. Overall, although HClust falls behind tfunHDDC in settings dominated by large functional covariance differences and outlying functions, it frequently achieves the highest clustering accuracy among the methods considered. In particular, HClust performs well when the clustering information is primarily contained in the mean structures, when differences in the mean and covariance structures are distributed across both the functional and vector components, or when the clustering signal arises from the covariance structure of the vector component.

To assess the sensitivity of these findings to the choice of weighting strategy discussed in Section 2.4, we conducted additional simulation studies using the alternative weighting approach, ω=|𝓣|/p. The results are presented in Table S1. Overall, the results obtained using the alternative weighting scheme (Table S1) are very similar to those obtained using the primary weighting scheme, which balances the observed total variability of the functional and vector components (Table 1). More specifically, the primary weighting scheme performs slightly better than the alternative weighting scheme in scenarios (i)–(iv), whereas the alternative weighting scheme performs slightly better for certain sample sizes in scenarios (v) and (vii). These minor differences do not materially affect the overall conclusions of the simulation study. In particular, the consistently similar performance under the two weighting schemes suggests that the proposed method is robust to reasonable variation in the relative weighting of the functional and vector components. Given this empirical robustness, together with the rationale described in Section 2.4 for balancing the observed total variability of the two components, we believe that the primary weighting scheme is appropriate for our setting.

We also considered scenarios with imbalanced cluster sizes, and the corresponding results are provided in the Supporting Information (Table S2). Specifically, we examined two additional sample-size configurations, (75,100) and (75, 125). As the degree of cluster-size imbalance increases, clustering accuracy tends to decrease across the methods considered. Nevertheless, HClust achieves the highest or near-highest accuracy in scenarios (i)–(iv) and (vi), broadly consistent with the findings under balanced cluster sizes. In scenario (v), HClust again does not outperform tfunHDDC, consistent with the balanced-sample results and with the favorable performance of tfunHDDC in settings characterized by large inter-cluster differences in functional covariance structure. In scenario (vii), HClust also performs below tfunHDDC under the more highly imbalanced configuration, suggesting that its relative advantage may be reduced when both the clustering signals and the representation of one cluster are limited. Overall, these results indicate that HClust remains competitive under imbalanced cluster sizes, while its relative advantage is more apparent in settings with balanced or moderately imbalanced clusters.

### 3.2 | Setup 2: Multivariate Functional Data (K=2; d=1) and Four-Dimensional Vector Data (p=4)

In this setup, we considered a hybrid object consisting of multivariate functional data with two functions (K=2) combined with 4-dimensional vector data (p=4). Two clusters c∈{1,2} of hybrid data were generated based on model 10, where the cluster-specific mean structure of functional signals, $F_{i}^{\left[c\right]}=(F_{i}^{\left(1)[c\right]},F_{i}^{\left(2)[c\right]})$, were specified as follows. For the large inter-cluster differences in the mean and covariance structures of the functional component, we set $\mu_{F}^{\left(1)[1\right]}(t)=0$ for all $t\in {𝓣}_{\text{grid}}$, $\mu_{F}^{\left(1)[2\right]}(t)=2{\left(t+0.5\right)}^{2}+t,\mu_{F}^{\left(2)[1\right]}(t)=0$, and $\mu_{F}^{\left(2)[2\right]}(t)=\sin(20{\left(t-1\right)}^{2})$, with $\Sigma {\left(s,t\right)}^{\left(1)[1\right]}=e^{-2{\left(s-t\right)}^{2}},\Sigma {\left(s,t\right)}^{\left(1)[2\right]}=0.8^{\left|s-t\right|}/\left(1-0.8^{2}\right)$, and $\Sigma {\left(s,t\right)}^{\left(2)[1\right]}=\Sigma {\left(s,t\right)}^{\left(2)[2\right]}=0.1^{\left|s-t\right|}/\left(1-0.1^{2}\right)$, for $s,t\in {𝓣}_{\text{grid}}$. For the small inter-cluster differences in the functional component, we set $\mu_{F}^{\left(1)[1\right]}(t)=4{\left(t-0.5\right)}^{2},\mu_{F}^{\left(1)[2\right]}(t)=1.5e^{\left(-20{\left(t-0.2\right)}^{2}\right)}+1.5e^{\left(-40{\left(t-0.2\right)}^{2}\right)},\mu_{F}^{\left(2)[1\right]}(t)=0.4{\left(t-0.5\right)}^{2}$, and $\mu_{F}^{\left(1)[2\right]}(t)=\sin{\left(15(t-1\right)}^{2}$), $\Sigma {\left(s,t\right)}^{\left(1)[1\right]}=e^{-2{\left(s-t\right)}^{2}},\Sigma {\left(s,t\right)}^{\left(1)[2\right]}=\Sigma {\left(s,t\right)}^{\left(2)[1\right]}=\Sigma {\left(s,t\right)}^{\left(2)[2\right]}=e^{-1.5{\left(s-t\right)}^{2}}$, for $s,t\in {𝓣}_{\text{grid}}$. For the vector component, we considered the same cluster-specific mean and covariance structures for the large and small inter-cluster difference settings as those described in Section 3.1. Because FClust and tfunHDDC are designed for univariate functional data, we applied these methods to the multivariate functional observations after concatenating the two functional components into a single functional object.

Table 2 presents the clustering accuracy of HClust and the competing methods across seven simulation scenarios, each replicated 100 times, with balanced sample sizes between clusters 1 and 2 (75, 100, or 125 subjects per cluster). In scenario (i), where the inter-cluster differences are large in both the functional and vector components, all methods perform well, with HClust, K-means-Vector, and HPCA achieving accuracy rates close to 1. In scenario (ii), where the inter-cluster differences in the functional component remain large but those in the vector component are small, HClust achieves the best performance by effectively incorporating the information from the functional component, whereas K-means-Vector, which relies solely on the vector component, performs poorly. In scenario (iii), where the inter-cluster differences in the functional component are small but those in the vector component are large, HClust, K-means-Vector, and HPCA achieve the best performance, whereas the functional-data-based methods, FClust and tfunHDDC, perform poorly.

In scenario (iv), where the inter-cluster differences are driven primarily by the mean structures of both components and the covariance differences are small, HClust again achieves the best performance, together with HPCA. This indicates that HClust performs well in settings driven primarily by mean differences, despite also accounting for covariance differences. In scenario (v), where the primary inter-cluster difference arises from the covariance structure of the functional component and the simulated data contain many outlying functions, HClust performs less favorably and falls behind K-means-Vector and HPCA. Unlike the corresponding univariate functional setting, tfunHDDC also performs poorly in this scenario, possibly because its application requires concatenation of the two functional components into a single functional object and therefore may not adequately preserve the multivariate functional structure. In scenario (vi), where the inter-cluster difference arises primarily from the covariance structure of the vector component, HClust achieves the best performance. HClust also performs best in scenario (vii), where the inter-cluster differences in both the mean and covariance structures are small for both the functional and vector components. Overall, these results indicate that HClust generally performs well for hybrid data with multivariate functional components, with performance patterns broadly consistent with those observed for hybrid data with univariate functional components.

### 3.3 | Setup 3: Univariate Image Data (K=1;d=2) and Two-Dimensional Vector Data (p=2)

In this setup, we consider clustering a single univariate (K= 1) two-dimensional function/image (d=2) and vector data consisting of two scalar variables (p=2). Data are generated under the model specified in Equation (10), with some modifications to adapt for image data. The cluster-specific functional signals, $F_{i}^{\left(1)[c\right]}$, are generated from a Gaussian Random Field (GRF) on an equally-spaced 10×10 grid with coordinates $\left(t_{1},t_{2}\right)\in \{1,\ldots ,10\}\times \{1,\ldots ,10\}$. The cluster-specific mean function is defined as $\mu_{F}^{\left(1)[c\right]}\left(t_{1},t_{2}\right)=\mu_{0,c}+\left(t_{1}^{2}-t_{2}^{2}\right)\cdot \left(\mu_{1,c}-\mu_{0,c}\right)$, where $\mu_{0,c}$ and $\mu_{1,c}$ are cluster-specific constants specified under different simulation scenarios. The covariance function is given by $C\left(t_{1},t_{2};t_{1}^{\prime },t_{2}^{\prime }\right)=\sigma_{c}^{2}\sqrt{t_{1}^{2}+t_{2}^{2}}\sqrt{t_{1}^{\prime 2}+t_{2}^{\prime 2}}exp(-h/0.01)$, where the cluster-specific variance term $\sigma_{c}^{2}$ is specified according to each simulation scenario, and h denotes the Euclidean distance between points ($t_{1},t_{2}$) and ($t_{1}^{\prime },t_{2}^{\prime }$). The cluster-specific vector signal $V_{i}^{\left[c\right]}=(V_{i}^{\left(1)[c\right]},V_{i}^{\left(2)[c\right]})$ is generated from a multivariate normal distribution $N_{2}(\mu_{V}^{\left[c\right]},I_{2})$, where $\mu_{V}^{\left[c\right]}=(\mu_{V}^{\left(1)[c\right]},\mu_{V}^{\left(2)[c\right]})$ is the cluster-specific mean vector specified according to simulation scenario. As in the other setups, a subject-specific random effect $W_{i}∼N(0,0.5)$ is generated and shared across both the functional and vector components.

We consider two clusters c∈{1,2} of data under two scenarios. The first scenario represents an easy, well-separated cluster case, where the cluster-specific constants for the mean function and variance terms in the GRF for the functional signals, and the mean vector in the multivariate normal distribution for the vector signals, are specified as follows: $\mu_{0,1}=190,\mu_{1,1}=192,\sigma_{1}^{2}=4,\mu_{0,2}=179,\mu_{1,2}=201,\sigma_{2}^{2}=6$, and $\mu_{X}^{c}=(5,15)$. The second scenario represents a difficult case with less separation between clusters, with parameters specified as: $\mu_{0,1}=180,\mu_{1,1}=185,\sigma_{1}^{2}=4,\mu_{0,2}=175,\mu_{1,2}=195,\sigma_{2}^{2}=6$, and $\mu_{X}^{c}=(5,10)$. Apart from our proposed method, HClust, we also applied the three competing methods examined in the first setup described in Section 3.1: FClust, tfunHDDC, and K-means-Both. Because the R packages for the first two methods cannot handle two-dimensional functional objects, the image data were vectorized to one-dimensional curves. K-means-Both was applied to the combined vectorized images and vector data as a single feature vector.

Table 3 presents the clustering accuracy of HClust and three existing methods across two simulation scenarios, each replicated 100 times, with balanced sample sizes between clusters 1 and 2 (50, 75, or 100 subjects per cluster). In the first scenario with well-separated clusters, the proposed method outperforms the other methods, except in the case of a cluster-specific sample size of 100, where HClust and K-means—both show identical performance. The largest discrepancies occur when the sample size is small, highlighting the ability of HClust to detect cluster structure even with limited information. In the second scenario with less-separated clusters, the performance of all methods declines, as expected, while HClust remains the top-performing method. These results indicate that HClust effectively identifies homogeneous subgroups in settings combining image and vector data, under both well-separated and poorly separated cluster conditions.

## 4 | Application to Renal Study

In this section, we apply the proposed method and demonstrate its utility using the Emory renogram data briefly described in Section 1. The dataset come from 253 kidneys from 131 patients who were referred to the clinic at Emory University Hospital with suspected kidney obstruction between January 1998 and July 2008. For each kidney, the data have a hybrid structure consisting of two renogram curves and six pharmacokinetic variables. The baseline renogram curve (*Y*^(1)^) represents time-varying MAG3 photon counts collected at 59 time points (every 16–30 s) during a 24-min scan, and the diuretic renogram curve (*Y*^(2)^) represents counts at 40 time points (every 30 s) during a subsequent 20-min scan following intravenous furosemide injection. The six scalar pharmacokinetic variables, which summarize key aspects of renal excretory and drainage function, are derived from the cortical renogram and include: area under the first derivative of the baseline curve (*X*^(1)^), minimum velocity of the baseline curve (*X*^(2)^), time to minimum velocity of the baseline curve (*X*^(3)^), time to maximum of the baseline curve (*X*^(4)^), area under the pelvis region of the diuretic curve (*X*^(5)^), and maximum of the pelvis region of the diuretic curve (*X*^(6)^). In sum, data collected for each kidney *i* ∈ {1, … , 253} take a hybrid form **Z***_i_* = (**Y***_i_* , **X***_i_*) = (*Y*^(1)^ , *Y*^(2)^ , *X*^(1)^ , … , *X*^(6)^). The left panel of Figure 1 displays standardized baseline renogram curves (first row) and standardized diuretic renogram curves (second row) for 50 selected kidneys, along with boxplots of the six pharmacokinetic variables (third row) based on all 253 kidneys.

To illustrate how renogram curves and pharmacokinetic variables inform the diagnosis of kidney obstruction, we plot data from seven kidneys for which obstruction status was determined by a nuclear medicine expert (four unobstructed and three obstructed). The right panel of Figure 1 presents standardized baseline (first row) and diuretic (second row) renogram curves, along with dot plots of the six pharmacokinetic variables (third row), stratified by obstruction status as determined by the nuclear medicine expert (green: unobstructed; red: obstructed). Some interpretive patterns emerge. Among the solid renogram curves, obstructed kidneys tend to exhibit prolonged MAG3 accumulation and delayed elimination during the diuretic phase, whereas unobstructed kidneys generally display rapid uptake and prompt elimination with near-complete clearance during diuresis. Also, higher values of the pharmacokinetic variables (circles) are associated with a greater likelihood of obstruction. An interesting exception occurs in one unobstructed kidney whose dotted renogram curves closely resemble those of obstructed kidneys. In this case, its similarity to the unobstructed group arises primarily from its pharmacokinetic variables (triangles), particularly the last three variables $\left(X^{\left(4\right)}-X^{\left(6\right)}\right)$. This example underscores both the substantial kidney-to-kidney variability observed in practice and the importance of jointly considering functional (renogram curves) and scalar (pharmacokinetic) data. Reliance on only one modality can lead to erroneous interpretation.

Taken together, these observations highlight the complexity of renogram interpretation involving two distinct data modalities and point to wider challenges in clinical practice. Interpreting renogram curves and pharmacokinetic variables effectively requires substantial expertise in renal physiology and MAG3 pharmacokinetics. Given that approximately 590,000 radionuclide renal scans are performed annually in the United States, most interpretations are conducted by general radiologists who typically receive only 3–4 months of nuclear medicine training, resulting in limited diagnostic accuracy (Taylor and Garcia, 2014; Jakšić et al., 2005). A major challenge for supporting sound interpretation and improving diagnostic accuracy, especially in settings with limited training time and high scan volume, is the absence of an established gold standard for obstruction diagnosis using radionuclide imaging. Expert stratification, as shown in the right panel of Figure 1, provides useful guidance but remains imperfect, and the small number of experts relative to the scan volume is insufficient to provide comprehensive and timely support for radiologists in practice. Given the lack of a clear gold standard, our goal is to identify homogeneous subgroups in a data-driven manner by jointly analyzing renogram curves and pharmacokinetic variables in a hybrid framework. This approach aims to define clinically meaningful risk groups that can support more targeted clinical management and decision-making.

We applied the proposed clustering method for hybrid data to the Emory renal study dataset. To determine the optimal number of clusters, we first used our data-driven approach described in Section 2.6. The corresponding jump plot, with the y-axis representing the “jumps” $\left(J_{C}\right)$ and the x-axis representing the number of clusters (C=1,2,…,10), is presented in Figure 2. Here, the transformation power B was selected to explain 80% of the variation in $\eta_{i}$ used in the distortion function formula (9). The plot shows the largest jump occurring at C=2 (represented by $J_{2}$), suggesting that the most significant improvement in cluster separation occurs with two clusters, which we chose as the optimal number. Accordingly, we applied our proposed algorithm to identify two clusters of hybrid renogram data encompassing baseline and diuretic renogram curves (functional data) and six pharmacokinetic variables (vector data) from 253 kidneys. We used the weight ω specified in Equation (8), which balances the total variability contributed by the functional component with the weighted total variability contributed by the vector component.

Figure 3 presents the results of the cluster analysis, which partitioned the data into two groups: 188 kidneys in Cluster 1 and 65 kidneys in Cluster 2, with cluster centers and clustered data plotted in green and red, respectively. The top panel shows the cluster centers (bold lines) for baseline (left) and diuretic (right) renogram curves, along with curves from 20 randomly selected kidneys from each cluster (thin lines). The bottom panel displays box plots of the six pharmacokinetic variables, stratified by cluster. We see that the two clusters closely correspond to patterns of renogram data with differing obstruction status as determined by the nuclear medicine expert (see right panel of Figure 1). Specifically, renogram data assigned to Cluster 1 exhibit a relatively short time to peak and rapid clearance of MAG3 to the bladder at baseline, followed by steady low levels during the diuretic (post-furosemide) phase, and relatively low levels of the pharmacokinetic variables, reflecting patterns observed in non-obstructed kidneys as determined by the nuclear medicine expert in Figure 1. In contrast, renogram data assigned to Cluster 2 show a prolonged time to peak at baseline, delayed clearance of MAG3 beginning in the diuretic phase, and relatively high levels of the pharmacokinetic variables, displaying characteristics observed in obstructed kidneys as determined by the nuclear medicine expert in Figure 1. These results demonstrate the utility of the proposed clustering method for identifying informative subgroups of hybrid renogram data and show its potential for supporting data-driven clinical interpretation and decision-making in settings where diagnostic errors are common due to limited radiologist experience and the absence of a clear gold standard.

## 5 | Discussion

Outcomes involving diverse data types are becoming increasingly common in clinical research. In this paper, we present a novel approach that integrates functional curves and vector data to cluster subjects into homogeneous groups. The proposed approach iteratively cycles through three steps: (i) extraction of cluster-specific mean and covariance structure from functional curves and vector data in a parsimonious manner based on HPCA; (ii) reconstruction of observations based on a cluster-specific KL expansion that captures cluster-specific mean and covariance structure; and (iii) assignment of cluster membership based on a hybrid distance between the raw and reconstructed hybrid observations. Our approach does not rely on parametric assumptions. Furthermore, application of the proposed clustering method to the renal study revealed subgroups of renogram curves and pharmacokinetic variables associated with kidney obstruction risk, demonstrating its scalability and potential for integration into a wide range of clinical studies collecting multiple data types.

Several limitations should be acknowledged, and opportunities for future research should be considered. First, although our method successfully integrates functional and vector components, it treats these two data types jointly through a common principal component framework without explicitly modeling potential complex interactions or dependencies between them. Future extensions could incorporate models that more explicitly account for these dependencies, possibly improving clustering performance when such interactions are important. Second, our method is distance-based and does not directly incorporate uncertainty quantification or probabilistic assessment of cluster membership, which could be desirable in clinical contexts where interpretability and decision support are critical. Bayesian formulations or soft-clustering extensions may help address this limitation. Third, while we demonstrated the scalability of our approach using one-dimensional curve and two-dimensional image data combined with vector data containing a moderate number of scalar elements, through simulations and a real analysis of renal imaging data, further evaluation in larger-scale settings involving three- or higher-dimensional functional objects (e.g., tensors) or ultra-high-dimensional vector data (p≫n) is warranted.

The proposed HClust algorithm involves additional computational cost because its iterative updating procedure uses leave-one-curve-out prediction to evaluate cluster membership based on cluster-specific hybrid mean–covariance structures. This additional computation is an intentional feature of the method, as it enables the clustering procedure to account for both mean differences and cluster-specific modes of functional–vector variation, rather than relying solely on a single clustering of marginal HPCA scores. In practice, the computational burden was manageable for the settings considered in this study. For example, in simulation scenario (i) with 75 observations per cluster, one iteration required approximately 0.36 min on average per simulated data set on a MacBook Pro with an Apple M4 chip. In the renal imaging application, which included *n* = 253 subjects with 59 baseline and 40 diuretic time points per curve, the algorithm converged after 12 iterations and required approximately 26.26 min in total on the same computing platform. These results suggest that the proposed method is computationally feasible for moderate-sized hybrid functional and vector datasets, while providing the flexibility needed to identify subgroups characterized by distinct mean and covariance structures.

## Supplementary Material

Supplement

Supplement 2

Additional supporting information can be found online in the Supporting Information section.

## Figures and Tables

**FIGURE 1 |:**
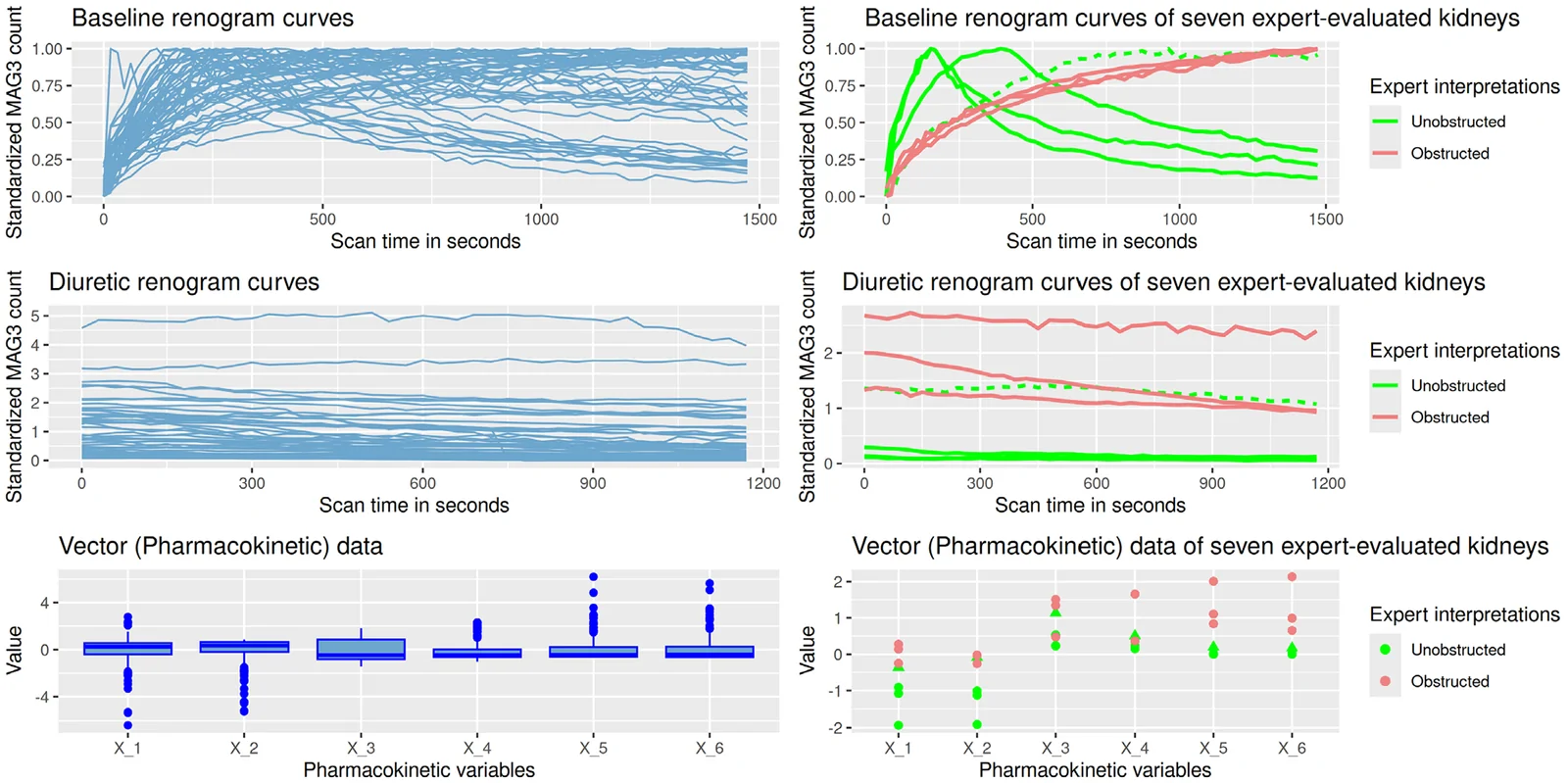
The left panel of the figure summarizes the hybrid data collected from 253 kidneys in the Emory renal study dataset. The first two rows of this panel display the baseline and diuretic renogram curves for 50 randomly selected kidneys, while the third row presents box plots of six pharmacokinetic variables derived from the cortical renograms of all 253 kidneys. The right panel shows baseline (first row) and diuretic (second row) renogram curves, together with the six pharmacokinetic variables (third row), for seven kidneys whose obstruction status was evaluated by a nuclear medicine expert and stratified accordingly (green: four unobstructed kidneys; red: three obstructed kidneys).

**FIGURE 2 |:**
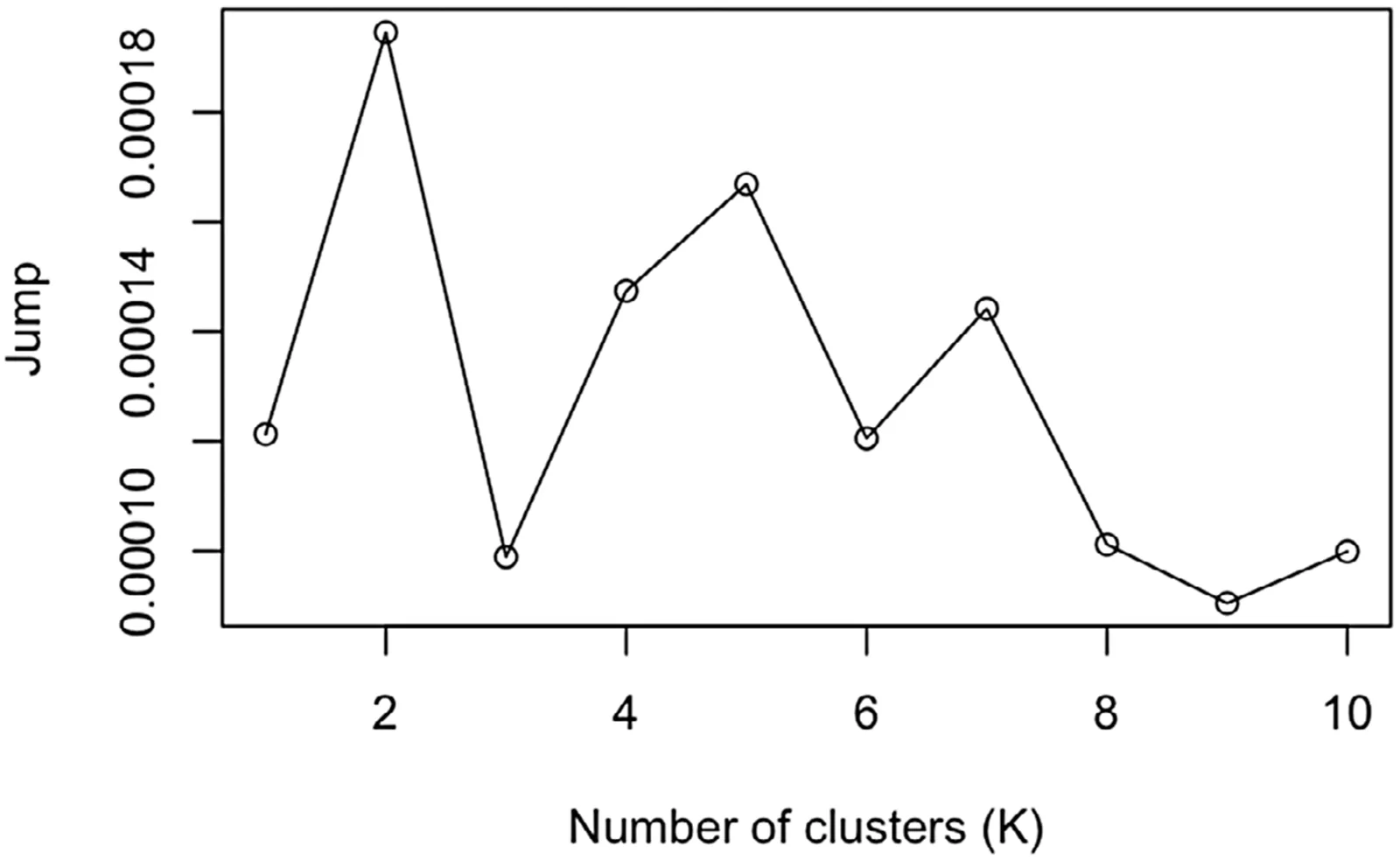
Jump plot for the renogram data, with the *y*-axis showing the “jumps” (*J_C_*) and the *x*-axis representing the varying number of clusters (*C*) over which the jumps are calculated. Each *J_C_* quantifies the reduction in distortion when increasing the number of clusters from *C* − 1 to *C*. Large jumps indicate significant improvements in cluster separation when adding additional clusters, while small jumps suggest diminishing returns. The transformation power *B* was selected to explain 80% of the variation in ***η**_i_*, which is used in the distortion formula (9).

**FIGURE 3 |:**
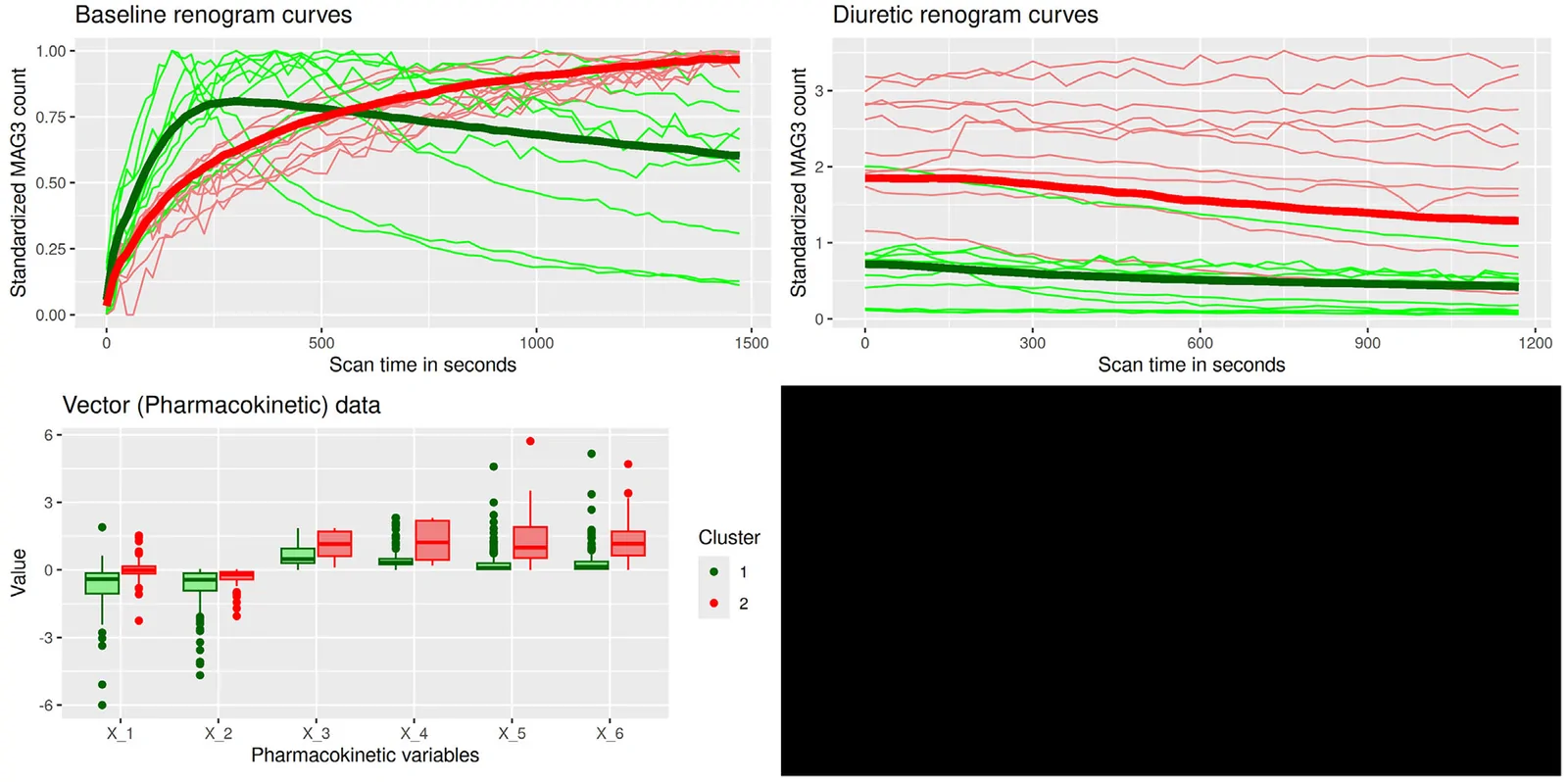
The two clusters of renogram data from 253 kidneys identified by the proposed clustering method for hybrid data (188 kidneys in Cluster 1 and 65 kidneys in Cluster 2). The top panel shows the clustered baseline (green) and diuretic (red) renogram curves, with green thin lines representing 20 randomly selected curves from Cluster 1 and red thin lines representing 20 randomly selected curves from Cluster 2; bold lines depict the respective cluster centers. The bottom panel presents box plots of the six pharmacokinetic variables, stratified by the two cluster groups.

**TABLE 1 |:** Clustering accuracy of HClust and competing methods across seven simulation scenarios (i)–(vii) representing varying inter-cluster differences in the mean and covariance structures of the functional and vector components (second to fifth columns). Data consist of univariate one-dimensional functional observations (*K* = 1, *d* = 1) and four-dimensional vector observations (*p* = 4); results are averaged over 100 replicates. FClust (Chiou and Li, 2007) and tfunHDDC (Schmutz et al., 2020) use only the functional component; Kmeans-Vector uses only the vector component; Kmeans-Both applies K-means to the concatenated vectorized functional and vector data; and HPCA clusters the HPCA scores (Jang, 2021). Cluster size (*a*, *b*) denotes the numbers of subjects in the two clusters generated under model (10).

Sce.	Function		Vector		Cluster size	HClust	FClust	tfun HDDC	Kmeans Vector	Kmeans Both	HPCA
	Mean	Var	Mean	Var							
(i)	Large	Large	Large	Large	(75,75)	1.0000	0.9811	0.8972	1.0000	0.9587	0.9999
					(100,100)	0.9997	0.9828	0.8673	1.0000	0.9569	0.9999
					(125,125)	0.9997	0.9826	0.8459	1.0000	0.9570	0.9999
(ii)	Large	Large	Small	Small	(75,75)	0.9877	0.9811	0.8972	0.6732	0.9557	0.9608
					(100,100)	0.9893	0.9828	0.8673	0.6886	0.9542	0.9596
					(125,125)	0.9881	0.9826	0.8459	0.6874	0.9537	0.9588
(iii)	Small	Small	Large	Large	(75,75)	1.0000	0.5936	0.6231	1.0000	0.7993	1.0000
					(100,100)	1.0000	0.5644	0.8712	1.0000	0.7691	1.0000
					(125,125)	1.0000	0.5854	0.8012	1.0000	0.7804	1.0000
(iv)	Large	Small	Large	Small	(75,75)	0.9999	0.9558	0.9825	1.0000	0.9487	0.9999
					(100,100)	0.9996	0.9576	0.9861	1.0000	0.9472	0.9999
					(125,125)	0.9997	0.9589	0.9883	1.0000	0.9478	0.9999
(v)	Small	Large	Small	Small	(75,75)	0.7527	0.7051	0.9208	0.6732	0.7053	0.7477
					(100,100)	0.7566	0.7048	0.9266	0.6887	0.6997	0.7473
					(125,125)	0.7559	0.7161	0.9267	0.6874	0.6997	0.7506
(vi)	Small	Small	Small	Large	(75,75)	0.7216	0.6687	0.6597	0.6619	0.6767	0.7066
					(100,100)	0.7143	0.6533	0.6659	0.6795	0.6731	0.7009
					(125,125)	0.7124	0.6459	0.6614	0.6824	0.6726	0.7018
(vii)	Small	Small	Small	Small	(75,75)	0.8097	0.5936	0.6231	0.6732	0.7739	0.7540
					(100,100)	0.8478	0.5644	0.8712	0.6886	0.7568	0.8321
					(125,125)	0.8816	0.5854	0.8012	0.6874	0.7600	0.8677

**TABLE 2 |:** Clustering accuracy of HClust and competing methods across seven simulation scenarios (i)–(vii) representing varying inter-cluster differences in the mean and covariance structures of the functional and vector components (second to fifth columns). Data consist of multivariate one-dimensional functional components (*K* = 2, *d* = 1) and a four-dimensional vector component (*p* = 4); results are averaged over 100 replicates. FClust (Chiou and Li, 2007) and tfunHDDC (Schmutz et al., 2020) use only the functional component; Kmeans-Vector uses only the vector component; Kmeans-Both applies K-means to the concatenated vectorized functional and vector data; and HPCA clusters the HPCA scores (Jang, 2021). Cluster size (*a*, *b*) denotes the numbers of subjects in the two clusters generated under model (10).

Sce.	Function		Vector		Cluster size	HClust	FClust	tfun HDDC	Kmeans Vector	Kmeans Both	HPCA
	Mean	Var	Mean	Var							
(i)	Large	Large	Large	Large	(75,75)	0.9979	0.9335	0.8359	1.0000	0.9707	1.0000
					(100,100)	0.9979	0.9200	0.8376	1.0000	0.9737	1.0000
					(125,125)	0.9978	0.9034	0.8383	1.0000	0.9710	1.0000
(ii)	Large	Large	Small	Small	(75,75)	0.9973	0.9335	0.8359	0.6843	0.9664	0.9636
					(100,100)	0.9977	0.9200	0.8376	0.6804	0.9699	0.9633
					(125,125)	0.9974	0.9034	0.8383	0.6909	0.9662	0.9626
(iii)	Small	Small	Large	Large	(75,75)	1.0000	0.5637	0.7321	1.0000	0.8411	1.0000
					(100,100)	1.0000	0.5534	0.7396	1.0000	0.8488	1.0000
					(125,125)	1.0000	0.5503	0.7436	1.0000	0.8506	1.0000
(iv)	Large	Small	Large	Small	(75,75)	1.0000	0.9892	0.8769	1.0000	0.9721	1.0000
					(100,100)	1.0000	0.9895	0.9003	1.0000	0.9749	1.0000
					(125,125)	1.0000	0.9872	0.8903	1.0000	0.9735	1.0000
(v)	Small	Large	Small	Small	(75,75)	0.7156	0.5668	0.6164	0.6843	0.7637	0.8367
					(100,100)	0.7578	0.5581	0.6302	0.6804	0.7566	0.8529
					(125,125)	0.7372	0.5461	0.6438	0.6909	0.7596	0.8514
(vi)	Small	Small	Small	Large	(75,75)	1.0000	0.5619	0.7008	0.6711	0.7542	0.8173
					(100,100)	1.0000	0.5636	0.7402	0.6728	0.7419	0.8408
					(125,125)	1.0000	0.5559	0.7281	0.6906	0.7428	0.8355
(vii)	Small	Small	Small	Small	(75,75)	1.0000	0.5637	0.7321	0.6843	0.8047	0.8617
					(100,100)	1.0000	0.5534	0.7395	0.6804	0.8006	0.9120
					(125,125)	1.0000	0.5503	0.7436	0.6909	0.7992	0.9200

**TABLE 3 |:** The accuracy rates of the proposed method (HClust) and competing methods across two simulation scenarios (well- vs. less-separated clusters) involving univariate two-dimensional (*K* = 1, *d* = 2) functional data and two-dimensional (*p* = 2) vector data, averaged over 100 replicates. “FClust” and “tfunHDDC” denote the functional clustering algorithms proposed by Chiou and Li (2007) and Schmutz et al. (2020), respectively, and are applied only to the functional component of the hybrid data, after vectorizing the image data to form a one-dimensional curve. “K-means-Both” refers to the conventional K-means algorithm applied after vectorizing the functional data and concatenating it with the vector data to form a single feature vector. The cluster size (*a*, *b*) indicates the number of subjects in each of the two clusters generated under model (10).

Scenario	Cluster size	HClust	FClust	tfun HDDC	Kmeans Both
Well-separated clusters	(50, 50)	0.9383	0.5678	0.8474	0.8417
	(75, 75)	0.9149	0.8345	0.8125	0.8804
	(100, 100)	0.8923	0.8478	0.8152	0.8974
Less-separated clusters	(50, 50)	0.6927	0.5371	0.6893	0.6757
	(75, 75)	0.7237	0.6231	0.6766	0.6904
	(100, 100)	0.7405	0.6442	0.6705	0.7026

## Data Availability

The dataset that supports the findings of this study is proprietary and confidential, and cannot be shared with the general public for subsequent research purposes. The authors do not have the permission to publish or share the raw data.
