# Supplementary material for: Clustering Hybrid Functional and Vector Data
Source: Biom J. Author manuscript; Available in PMC 2026 Nov 16. (PMC13773016; doi:10.1002/bimj.70184)
Supplement: Supplementary file 1 [file NIHMS2218753-supplement-Supplement.pdf]

Web Supplement: "Clustering Hybrid Functional and Vector Data"

September 22, 2026

Table S1: Clustering accuracy of HClust and competing methods across seven simulation scenarios (i)-(vii) representing varying inter-cluster differences in the mean and covariance structures of the functional and vector components (second to fifth columns), under the second weighting scheme of $\omega = |\mathcal{T}|/p$ (Ramsay and Silverman, 2005). Data consist of a univariate one-dimensional functional component ($K = 1$, $d = 1$) and a four-dimensional vector component ($p = 4$); results are averaged over 100 replicates. FClust (Chiou and Li, 2007) and tfunHDDC (Schmutz et al., 2020) use only the functional component; Kmeans-Vector uses only the vector component; Kmeans-Both applies K-means to the concatenated vectorized functional and vector data; and HPCA clusters the HPCA scores (Jang, 2021). Cluster size (a, b) denotes the numbers of subjects in the two clusters generated under model (10).

Sce.	Function		Vector		Cluster size	HClust	FClust	tfun HDDC	Kmeans Vector	Kmeans Both	HPCA
	Mean	Var	Mean	Var							
(i)	Large	Large	Large	Large	(75,75)	0.9931	0.9811	0.8972	1.0000	0.9563	0.9831
					(100,100)	0.9934	0.9828	0.8674	1.0000	0.9546	0.9831
					(125,125)	0.9926	0.9826	0.8459	1.0000	0.9542	0.9822
(ii)	Large	large	Small	Small	(75,75)	0.9874	0.9811	0.8972	0.6732	0.9556	0.9577
					(100,100)	0.9892	0.9828	0.8674	0.6887	0.9542	0.9557
					(125,125)	0.9880	0.9826	0.8459	0.6874	0.9538	0.9555
(iii)	Small	Small	Large	Large	(75,75)	0.9821	0.5936	0.6231	1.0000	0.7798	0.9745
					(100,100)	1.0000	0.5644	0.8712	1.0000	0.7574	1.0000
					(125,125)	1.0000	0.5854	0.8012	1.0000	0.7724	1.0000
(iv)	Large	Small	Large	Small	(75,75)	0.9894	0.9558	0.9825	1.0000	0.9459	0.9772
					(100,100)	0.9876	0.9576	0.9861	1.0000	0.9437	0.9788
					(125,125)	0.9863	0.9589	0.9883	1.0000	0.9447	0.9770
(v)	Small	Large	Small	Small	(75,75)	0.7503	0.7051	0.9208	0.6732	0.7051	0.7418
					(100,100)	0.7580	0.7048	0.9266	0.6887	0.6997	0.7409
					(125,125)	0.7561	0.7161	0.9267	0.6874	0.6999	0.7460
(vi)	Small	Small	Small	Large	(75,75)	0.7239	0.6687	0.6597	0.6619	0.6770	0.7015
					(100,100)	0.7156	0.6533	0.6659	0.6795	0.6729	0.6943
					(125,125)	0.7146	0.6459	0.6614	0.6824	0.6726	0.6989
(vii)	Small	Small	Small	Small	(75,75)	0.7947	0.5936	0.6231	0.6732	0.7744	0.7244
					(100,100)	0.8529	0.5644	0.8712	0.6887	0.7568	0.8108
					(125,125)	0.8775	0.5854	0.8012	0.6874	0.7565	0.8453

Table S2: Clustering accuracy of HClust and competing methods across seven simulation scenarios (i)-(vii) representing varying inter-cluster differences in the mean and covariance structures of the functional and vector components (second to fifth columns). Data consist of a univariate one-dimensional functional component ($K = 1$, $d = 1$) and a four-dimensional vector component ($p = 4$); results are averaged over 100 replicates. FClust (Chiou and Li, 2007) and tfunHDDC (Schmutz et al., 2020) use only the functional component; Kmeans-Vector uses only the vector component; Kmeans-Both applies K-means to the concatenated vectorized functional and vector data; and HPCA clusters the HPCA scores (Jang, 2021). Cluster size (a, b) denotes the numbers of subjects in the two clusters generated under model (10); this table additionally includes results for two imbalanced cluster-size settings.

Sce.	Function		Vector		Cluster size	HClust	FClust	tfun HDDC	Kmeans Vector	Kmeans Both	HPCA
	Mean	Var	Mean	Var							
(i)	Large	Large	Large	Large	(75,75)	1.0000	0.9811	0.8972	1.0000	0.9587	0.9999
					(75,100)	0.8571	0.8429	0.7735	0.8571	0.8247	0.8571
					(75,125)	0.7500	0.7386	0.7078	0.7500	0.7238	0.7500
(ii)	Large	Large	Small	Small	(75,75)	0.9877	0.9811	0.8972	0.6732	0.9557	0.9608
					(75,100)	0.8476	0.8429	0.7735	0.6615	0.8223	0.8271
					(75, 125)	0.7419	0.7386	0.7078	0.6265	0.7224	0.7266
(iii)	Small	Small	Large	Large	(75,75)	1.0000	0.5936	0.6231	1.0000	0.7993	1.0000
					(75,100)	0.8571	0.6935	0.7262	0.8571	0.7271	0.8571
					(75,125)	0.7500	0.5448	0.6917	0.7500	0.6410	0.7500
(iv)	Large	Small	Large	Small	(75,75)	0.9999	0.9558	0.9825	1.0000	0.9487	0.9999
					(100,100)	0.8570	0.8267	0.8471	0.8571	0.8206	0.8570
					(125,125)	0.7500	0.7301	0.7420	0.7500	0.7244	0.7500
(v)	Small	Large	Small	Small	(75,75)	0.7527	0.7051	0.9208	0.6732	0.7053	0.7477
					(75,100)	0.6435	0.6350	0.7833	0.6615	0.6398	0.6748
					(75,125)	0.5706	0.5849	0.7074	0.6265	0.5917	0.6118
(vi)	Small	Small	Small	Large	(75,75)	0.7216	0.6687	0.6597	0.6619	0.6767	0.7066
					(75,100)	0.6618	0.6164	0.6249	0.6601	0.6278	0.6474
					(75,125)	0.6135	0.5828	0.6029	0.6294	0.5868	0.6022
(vii)	Small	Small	Small	Small	(75,75)	0.8097	0.5936	0.6231	0.6732	0.7739	0.7540
					(75,100)	0.7170	0.6935	0.7262	0.6615	0.7181	0.6846
					(75,125)	0.6363	0.5448	0.6917	0.6265	0.6280	0.5981

References

Chiou, J.-M. and Li, P.-L. (2007). Functional clustering and identifying substructures of longitudinal data. *Journal of the Royal Statistical Society Series B: Statistical Methodology*, 169(4):679–699.

Jang, J. H. (2021). Principal component analysis of hybrid functional and vector data. *Statistics in Medicine*, 40(24):5152–5173.

Ramsay, J. O. and Silverman, B. W. (2005). *Functional Data Analysis*. Springer.

Schmutz, A., Jacques, J., Bouveyron, C., Cheze, L., and Martin, P. (2020). Clustering multivariate functional data in group-specific functional subspaces. *Computational Statistics*, 35(3):1101–1131.
